# Deficiency in the endocytic adaptor proteins PHETA1/2 impairs renal and craniofacial development

**DOI:** 10.1242/dmm.041913

**Published:** 2020-05-26

**Authors:** Kristin M. Ates, Tong Wang, Trevor Moreland, Rajalakshmi Veeranan-Karmegam, Manxiu Ma, Chelsi Jeter, Priya Anand, Wolfgang Wenzel, Hyung-Goo Kim, Lynne A. Wolfe, Joshi Stephen, David R. Adams, Thomas Markello, Cynthia J. Tifft, Robert Settlage, William A. Gahl, Graydon B. Gonsalvez, May Christine Malicdan, Heather Flanagan-Steet, Y. Albert Pan

**Affiliations:** 1 Department of Neuroscience and Regenerative Medicine, Medical College of Georgia, Augusta University, Augusta, GA 30912, USA; 2 Center for Neurobiology Research, Fralin Biomedical Research Institute at Virginia Tech Carilion, Virginia Tech, Roanoke, VA 24016, USA; 3 JC Self Research Institute, Greenwood Genetic Center, Greenwood, SC 29646, USA; 4 Department of Cellular Biology and Anatomy, Medical College of Georgia, Augusta University, Augusta, GA 30912, USA; 5 Institute of Nanotechnology, Karlsruhe Institute of Technology, 76021 Karlsruhe, Germany; 6 Neurological Disorder Research Center, Qatar Biomedical Research Institute, Hamad Bin Khalifa University, Doha, Qatar; 7 Medical Genetics Branch, National Human Genome Research Institute, National Institutes of Health, Bethesda, MD 20892, USA; 8 Advanced Research Computing Unit, Division of Information Technology, Virginia Tech, Blacksburg, VA 24060, USA; 9 National Institutes of Health Undiagnosed Diseases Program, National Institutes of Health, Bethesda, MD 20892, USA; 10 Department of Biomedical Sciences and Pathobiology, Virginia-Maryland College of Veterinary Medicine, Virginia Tech, Blacksburg, VA 24060, USA; 11 Department of Psychiatry and Behavioral Medicine, Virginia Tech Carilion School of Medicine, Roanoke, VA 24016, USA

**Keywords:** PHETA1, IPIP27A, OCRL, Endocytosis, Undiagnosed disease

## Abstract

A critical barrier in the treatment of endosomal and lysosomal diseases is the lack of understanding of the *in vivo* functions of the putative causative genes. We addressed this by investigating a key pair of endocytic adaptor proteins, PH domain-containing endocytic trafficking adaptor 1 and 2 (PHETA1/2; also known as FAM109A/B, Ses1/2, IPIP27A/B), which interact with the protein product of *OCRL*, the causative gene for Lowe syndrome. Here, we conducted the first study of PHETA1/2 *in vivo*, utilizing the zebrafish system. We found that impairment of both zebrafish orthologs, *pheta1* and *pheta2*, disrupted endocytosis and ciliogenesis in renal tissues. In addition, *pheta1/2* mutant animals exhibited reduced jaw size and delayed chondrocyte differentiation, indicating a role in craniofacial development. Deficiency of *pheta1/2* resulted in dysregulation of cathepsin K, which led to an increased abundance of type II collagen in craniofacial cartilages, a marker of immature cartilage extracellular matrix. Cathepsin K inhibition rescued the craniofacial phenotypes in the *pheta1/2* double mutants. The abnormal renal and craniofacial phenotypes in the *pheta1/2* mutant animals were consistent with the clinical presentation of a patient with a *de novo* arginine (R) to cysteine (C) variant (R6C) of PHETA1. Expressing the patient-specific variant in zebrafish exacerbated craniofacial deficits, suggesting that the R6C allele acts in a dominant-negative manner. Together, these results provide insights into the *in vivo* roles of PHETA1/2 and suggest that the R6C variant is contributory to the pathogenesis of disease in the patient.

This article has an associated First Person interview with the first author of the paper.

## Abstract (precis)

**Editor's choice:** The functions of two endocytic adaptor proteins, PHETA1/2, are determined using zebrafish mutants and a potentially disease-causing variant of human PHETA1. Findings suggest essential roles in craniofacial and renal development.

## INTRODUCTION

Endocytic trafficking is essential for a variety of biological processes, including nutrient uptake, cell signaling and cellular morphogenesis (Doherty and McMahon, 2009). This diversity in cellular functions is reflected in the broad range of pathologies associated with deficiencies in endocytic factors. For example, mutations in endocytic factors dynamin 2 (*DNM2*) and *RAB7* result in Charcot-Marie-Tooth disease, a clinically and genetically heterogeneous group of peripheral neuropathies (Verhoeven et al., 2003; Züchner et al., 2005). Disruptions in endocytosis have been identified in autosomal recessive hypercholesterolemia (Garuti et al., 2005) and autosomal dominant polycystic kidney disease (Obermuller et al., 2001). These disparate clinical outcomes resulting from endocytic protein deficiency underscore the importance of investigations in the organismal context. Currently, endocytic pathways have been identified and defined through their differential interactions with specific phosphoinositides and proteins (e.g. clathrin, actin and dynamin), but most components of the endocytic machinery have only been examined in cell lines (Doherty and McMahon, 2009). In this study, our goal was to use an *in vivo* experimental system to investigate two important regulators of endocytosis, PH domain-containing endocytic trafficking adaptor 1 and 2 (PHETA1/2).

PHETA1/2 (also known as FAM109A/B, Ses1/2, IPIP27A/B) were identified *in vitro* as regulators of endosomal trafficking, specifically for receptor recycling to endosomes and for cargo sorting to lysosomes (Noakes et al., 2011; Swan et al., 2010). Both PHETA1 and PHETA2 have a C-terminal phenylalanine-histidine motif (F&H motif) that serves as a binding site for OCRL, encoded by a gene that is mutated in Lowe syndrome (MIM #309000) (Pirruccello et al., 2011). OCRL is an inositol 5-phosphatase, with phosphatidylinositol 4,5-bisphosphate [PI(4,5)*P_2_*] as the preferred substrate (Attree et al., 1992; Noakes et al., 2011). Binding to PI(4,5)*P_2_* occurs at the pleckstrin homology (PH) domain in OCRL, which also contains a loop outside the domain fold with a clathrin-binding motif. This motif directs OCRL specifically to clathrin-coated endocytic pits on the plasma membrane (Choudhury et al., 2009; Mao et al., 2009). PI(4,5)*P_2_* is abundant at the plasma membrane and is involved in a wide variety of processes, including actin dynamics and endocytosis (Sasaki et al., 2009). Disrupting the phosphatase activity of OCRL interferes with PI(4,5)*P_2_* homeostasis, which is thought to contribute to the disease manifestations of Lowe syndrome (De Leo et al., 2016; Lowe, 2005; Vicinanza et al., 2011).

Several studies have shown that PHETA1 is crucial in maintaining optimal OCRL function. Specifically, the 5-phosphatase activity of OCRL relies upon PHETA1-mediated interaction with protein kinase C and casein kinase substrate in neurons 2 (PACSIN2), a protein that interacts with the actin cytoskeleton. A proline-rich PPPxPPRR motif in PHETA1 located upstream of the F&H motif serves as the necessary PACSIN2 binding site (Billcliff et al., 2016). PHETA2 lacks the PPPxPPRR motif. OCRL also promotes ciliogenesis by way of endosomal trafficking in a Rab8 (also known as RAB8A)/PHETA1-dependent manner (Coon et al., 2012). These findings suggest that PHETA1 and OCRL functionally interact to mediate both endocytosis and ciliogenesis.

Besides endocytosis and ciliogenesis, PHETA1 and PHETA2 are also involved in the transport of newly synthesized lysosomal hydrolases from the trans-Golgi network (TGN) to the endosomes (Noakes et al., 2011). Thus, loss of PHETA1/2 could result in improper sorting of lysosomal hydrolases. Consistent with this idea, loss of PHETA2 results in hypersecretion of pro-cathepsin D (Noakes et al., 2011). Similar disruptions in lysosomal proteins have also been found in mucolipidosis type II (MLII), where the loss of mannose 6-phosphate-dependent targeting results in hypersecretion of multiple lysosomal enzymes (Kollmann et al., 2010; Kornfeld, 1986; Kudo et al., 2006). Dysregulation of cathepsins in MLII zebrafish models results in craniofacial and skeletal deformations, recapitulating the clinical features of MLII patients (Flanagan-Steet et al., 2009; Petrey et al., 2012). Thus, PHETA1/2-dependent regulation of protease transport may be important for craniofacial development.

The involvement of PHETA1 during development is further supported by recent findings in a human patient with a *de novo* arginine to cysteine (R6C) variant in PHETA1, identified through the National Institutes of Health (NIH) Undiagnosed Diseases Program (UDP) (Gahl et al., 2012, 2016, 2015). The UDP patient presented with global developmental delay, coarse facial features, renal abnormalities and other developmental deficits (Table 1). Despite the known roles of PHETA1 in facilitating OCRL function, the UDP patient did not present with the typical manifestations of Lowe syndrome, i.e. congenital cataracts, cognitive impairment, and renal tubular and glomerular dysfunction (Mehta et al., 2014). These findings suggest that PHETA1 may have OCRL-independent functions *in vivo*.

**Table 1.**
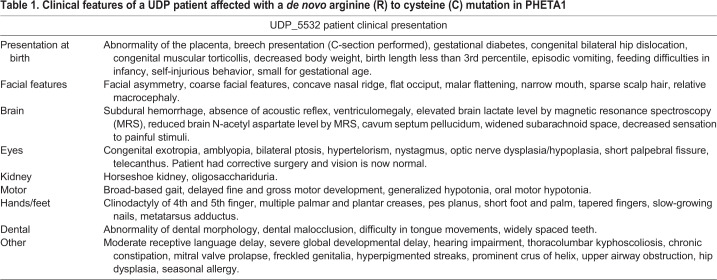
**Clinical features of a UDP patient affected with a *de novo* arginine (R) to cysteine (C) mutation in PHETA1**

To investigate the *in vivo* functions of PHETA1 and its close homolog PHETA2, we utilize zebrafish, an informative small vertebrate model organism for validating the pathogenicity of genes or alleles in human patients. This approach has offered valuable insight for clinicians into a broad range of genetic disorders, including neurodevelopmental disorders, ciliopathies and Lowe syndrome (Coon et al., 2012; Oltrabella et al., 2015; Phillips and Westerfield, 2014; Ramirez et al., 2012; Sakai et al., 2018; Song et al., 2016). Using histological, physiological and behavioral analyses, we found that zebrafish *pheta1* and *pheta2* are required for endocytosis, ciliogenesis and craniofacial development. Consistent with a role in trafficking lysosomal enzymes, disruption in craniofacial development in the *pheta1/2* mutants was associated with dysregulated cathepsin K activity. The abnormal craniofacial development is exacerbated further in the presence of the R6C variant, suggesting a dominant-negative mode of action in human disease.

## RESULTS

### Identification of a *de novo* PHETA1 variant in undiagnosed human disease

**Fig. 1.**
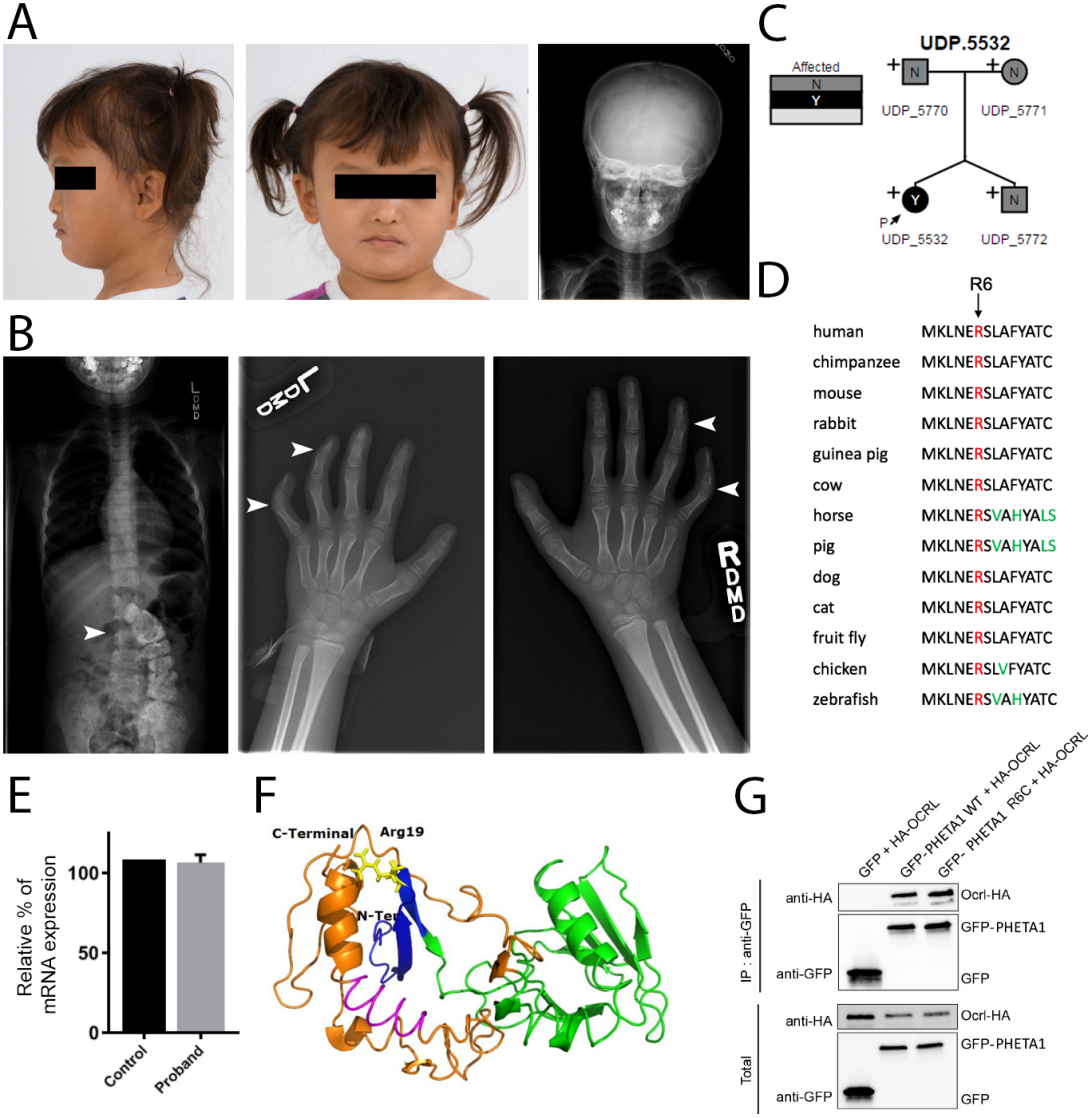
**Identification of a *de novo* mutation in human PHETA1.** (A) Images of the UDP patient, presenting with facial asymmetry, concave nasal ridge and malar flattening. Radiograph (right) reveals mild asymmetry of the skull. (B) Radiographs depict scoliosis (arrowhead in left image) and clinodactyly of fourth and fifth digits on both hands (arrowheads in middle and right images). (C) Whole-exome sequencing was performed on both parents and the fraternal twin of the UDP patient. ‘N’ denotes not affected and ‘Y’ denotes affected. The arrow labeled ‘P’ identifies the UDP patient (UDP_5532). ‘+’ indicates the presence of a normal allele, thus marking p.R6C as a heterozygous mutation. (D) COBALT multiple alignment of partial protein sequences of PHETA1 orthologs. The conserved arginine residue is highlighted in red, and amino acid residues that differ from the sequence of the human PHETA1 protein are highlighted in green. The arginine residue is highly conserved across multiple species. (E) Relative quantification of mRNA expression in the patient cells showing that the expression of *PHETA1* is not significantly different from that in controls. Error bar represents s.d. from six replicates. (F) 3-D structure of the human PHETA1 protein showing the PH domain (green) with a four-stranded N-terminal and three-stranded C-terminal β-sheet with a helix (orange). The conserved arginine amino acid (Arg19 in the PHETA1 long isoform, yellow) is far from the F&H motif (magenta); however, it stabilizes the folded domain around the C-terminal helix. (G) GFP-tagged full-length WT PHETA1 or PHETA1^R6C^ were expressed in HeLa cells and tested for interaction with full-length HA-tagged OCRL1. Bound proteins detected by western blotting with the indicated antibodies. ‘IP: anti-GFP’ refers to the anti-GFP antibody-bound fraction; ‘Total’ represents the total cell lysate.

The UDP enrolled a 6-year-old female patient with craniofacial dysmorphic features, scoliosis, clinodactyly, global developmental delay, vision and auditory impairments, and renal tubular or glomerular dysfunction (Fig. 1A,B and Table 1). Whole-exome sequencing and Sanger sequencing of the patient, unaffected fraternal twin and unaffected parents identified a heterozygous *de novo* arginine (R) to cysteine (C) mutation in *PHETA1*, present only in the patient (NM_001177997.2:c.55C>T; p.R6C in the short isoform; p.R19C in the long isoform, NM_001177996.1) (Fig. 1C). The R6 residue in PHETA1 is highly conserved across species (Fig. 1D) (Papadopoulos and Agarwala, 2007), and the R6C mutation was predicted to be damaging with the use of Polyphen (Probably damaging, HumDiv: 1; HumVar: 0.995), SIFT (Deleterious, Score 0.01), and MutationTaster (Disease causing, Prob: 1) (Adzhubei et al., 2010; Schwarz et al., 2014; Sim et al., 2012). This variant has been reported in the ExAC browser, with a minor allele frequency of 0.000009398 (1/106410). Using patient-derived fibroblasts and quantitative real-time PCR (qRT-PCR), we found that the R6C mutation does not affect the mRNA expression of *PHETA1* (Fig. 1E).

Protein modeling was performed using the I-TASSER, MUSTER and PHYRE2 servers (Fig. 1F) (Adzhubei et al., 2010; Kelley et al., 2015; Roy et al., 2010; Wu and Zhang, 2008). Based on the homology model, the arginine residue (highlighted in yellow) is far from the OCRL binding site (highlighted in magenta). However, it stabilizes the folded domain around the C-terminal helix, close to the F&H motif, such that the mutation is predicted to disrupt the folded domain and thus may interfere with OCRL binding to PHETA1. To test this, we expressed wild-type (GFP-PHETA1^WT^) and mutant (GFP-PHETA1^R6C^) GFP-tagged PHETA1 in HeLa cells, along with hemagglutinin (HA)-tagged OCRL. Surprisingly, we found that HA-OCRL was co-immunoprecipitated by both wild-type and mutant PHETA1 (Fig. 1G). This suggests that the R6C mutation might disrupt PHETA1 protein function in a manner that does not affect OCRL binding. Since the *in vivo* functions of PHETA1 and its close homolog, PHETA2, were still unknown, we used zebrafish as the experimental system to determine the roles of PHETA1/2 in the context of a vertebrate organism.

### Identification and CRISPR-Cas9 targeting of zebrafish *pheta1* and *pheta2*

**Fig. 2.**
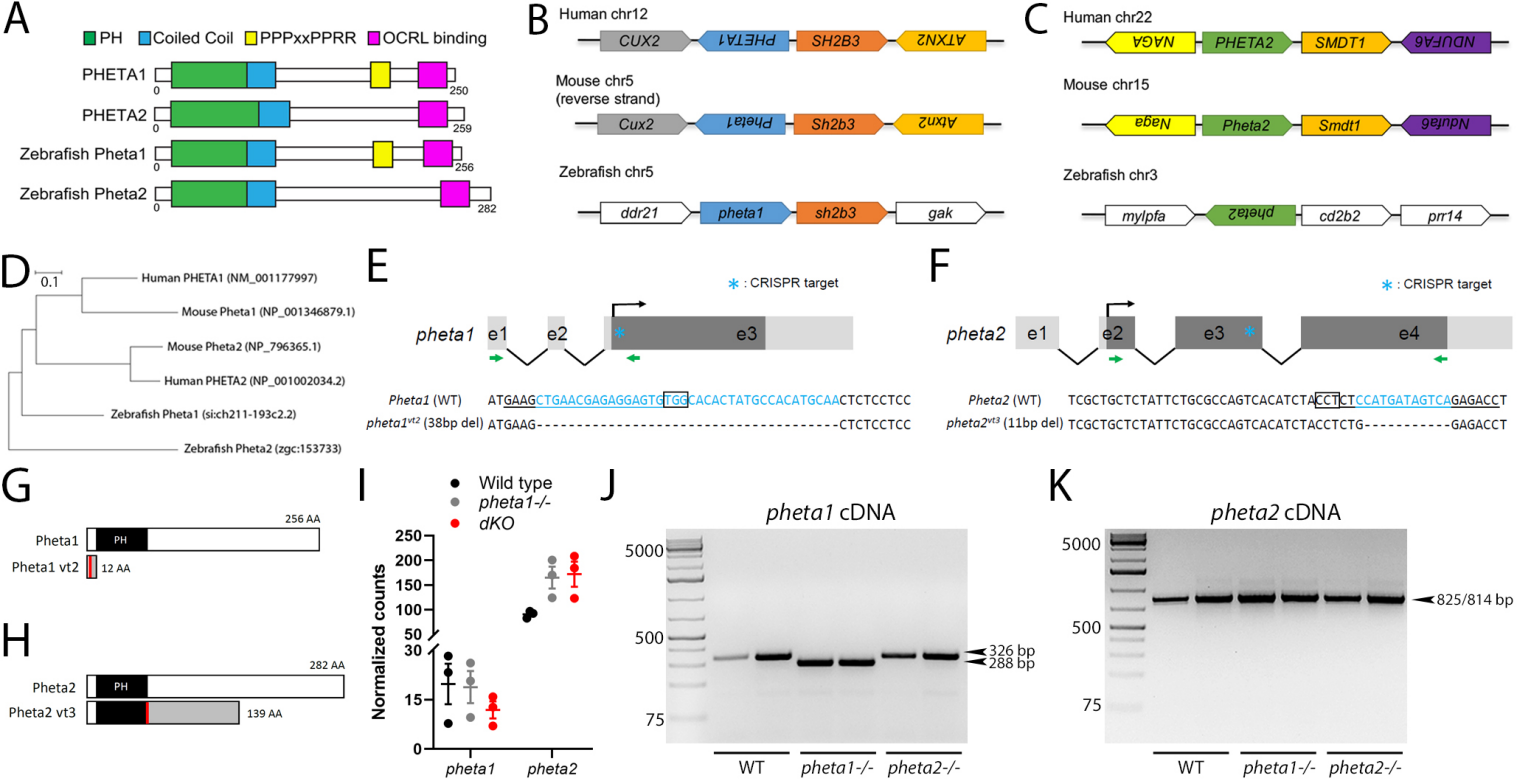
**Homology and CRISPR-Cas9 targeting of zebrafish *pheta1* and *pheta2*.** (A) The domain structures of human PHETA1, human PHETA2, and the zebrafish PHETA homologs Pheta1 and Pheta2. The PH domain, coiled-coil domain, PPPxPPRR motif and OCRL binding site are highlighted. Like human PHETA2, zebrafish Pheta2 lacks the PPPxPPRR motif. (B,C) Genomic organization of human *PHETA1* (B) and *PHETA2* (C) and their respective homologs in mouse and zebrafish. (D) Phylogenic tree of PHETA proteins. Units indicate the number of amino acid substitutions per site. (E,F) The diagrams on the top illustrate the genomic structures of *pheta1* and *pheta2*, with the guide RNA (gRNA) target marked with the asterisk. Green arrows indicate the primers for RT-PCR. Sequences at the bottom show the gRNA target (underlined) associated with the protospacer adjacent motif (PAM; boxed letters), and the sequences mutated by CRISPR-Cas9 (blue). (G,H) CRISPR-mediated mutations result in reading frame shifts, causing aberrant protein sequences (gray regions) and premature termination of the protein sequences for Pheta1 (G) and Pheta2 (H). The start of frameshift mutation is indicated by the red lines. (I) Normalized counts of *pheta1* and *pheta2* transcripts. (J,K) RT-PCR amplification of *pheta1* (J) and *pheta2* (K) complementary DNA (cDNA) from 4 dpf WT, *pheta1^−/−^* and *pheta2^−/−^* animals, using the primer pairs indicated in E and F. Two lanes from two separate pools of animals are shown for each genotype. No alternative splice forms were detected in the mutants. Expected sizes for *pheta1*: 326 bp (WT and *pheta2^−/−^*) and 288 bp (*pheta1^−/−^*)*.* Expected sizes for *pheta2*: 825 bp (WT and *pheta1^−/−^*) and 814 bp (*pheta2^−/−^*).

We identified two PHETA1/2 homologs in zebrafish using *in silico* sequence searches (see Materials and Methods). We refer to them as Pheta1 (encoded by *si:ch211-193c2.2*) and Pheta2 (encoded by *zgc:153733*). All known protein domain structures are conserved between human and zebrafish proteins, including the F&H motif (site of OCRL binding) (Fig. 2A). Like human PHETA1, Pheta1 (but not Pheta2) contains the PPPxPPRR motif for PACSIN2 binding. The neighboring genes of the human *PHETA1* (*SH2B3*) and zebrafish *pheta1* (*sh2b3*) are also homologous, indicating that the loci are syntenic and suggesting that zebrafish *pheta1* is the most likely ortholog of *PHETA1* (Fig. 2B). The zebrafish *pheta2* locus lacked obvious synteny with either the human *PHETA1* or *PHETA2* loci (three neighboring genes examined; Fig. 2C). Based on overall amino acid sequence similarity and phylogenetic distance (see Materials and Methods), zebrafish Pheta1 is more similar to mammalian PHETA1/2 (57.3% and 48.9% similarity to human PHETA1 and PHETA2, respectively), whereas Pheta2 is more divergent (44.2% and 39.2% similarity, respectively) (Fig. 2D).

To determine the functions of *pheta1* and *pheta2 in vivo*, we generated mutant alleles of *pheta1* and *pheta2* utilizing CRISPR-Cas9 genome engineering (Gagnon et al., 2014; Jao et al., 2013) (Fig. 2E). Genomic DNA sequencing showed that the *pheta1^vt2^* allele contains a 38 bp deletion after the start codon of *pheta1*, resulting in frameshift and predicted premature translational termination, suggesting that this is likely a null allele (Fig. 2E,G). The *pheta2^vt3^* allele contains an 11 bp deletion in exon 3 of *pheta2*, also resulting in a frameshift and predicted premature translational termination, suggesting that this is also a null allele (Fig. 2F,H). Sequencing of purified cDNA from *pheta1^vt2^* and *pheta2^vt3^* homozygous animals (*pheta1^−/−^* and *pheta2^−/−^*) confirmed the incorporation of the 38 bp and 11 bp deletion in the *pheta1* and *pheta2* transcript, respectively. Using RNA sequencing (RNA-seq), we found no significant differences in the amount of *pheta1* or *pheta2* transcripts among wild-type (WT), *pheta1^−/−^* or double mutant (*dKO*) animals, suggesting that the CRISPR-induced mutations do not induce nonsense-mediated decay of *pheta1/2* (Fig. 2I). RT-PCR with primers flanking the CRISPR-Cas9 target site showed that *pheta1* and *pheta2* mRNA were spliced correctly in the respective mutants, and no splice variants were detected (Fig. 2J,K).

Zygotic *pheta1^−/−^* and *pheta2^−/−^* animals were viable and fertile, with no external abnormalities during development (not shown). *pheta1* and *pheta2* transcripts were detected from before zygotic genome activation (one-cell and 512-cell stages) and were present during early development [1-3 days post-fertilization (dpf)] (Fig. S1). The early expression indicates that *pheta1* and *pheta2* are maternally inherited, and the maternal transcript might compensate for the loss of zygotic transcripts during the early stages of development. We, therefore, focused on the maternal-zygotic *pheta1^−/−^*, *pheta2^−/−^* and *dKO* animals (progeny from homozygous mutant mothers), which lacked functional maternal and zygotic transcript during development. We found that maternal-zygotic *pheta1^−/−^*, *pheta2^−/−^* and *dKO* animals were viable and fertile as well, with no external abnormalities during development. Therefore, *pheta1* and *pheta2* are not required for viability and fertility in zebrafish. Maternal-zygotic mutant animals were used for all subsequent studies except for oculomotor function assays, in which zygotic mutants were tested.

### Loss of *pheta1* and *pheta2* impairs fluid-phase endocytosis

**Fig. 3.**
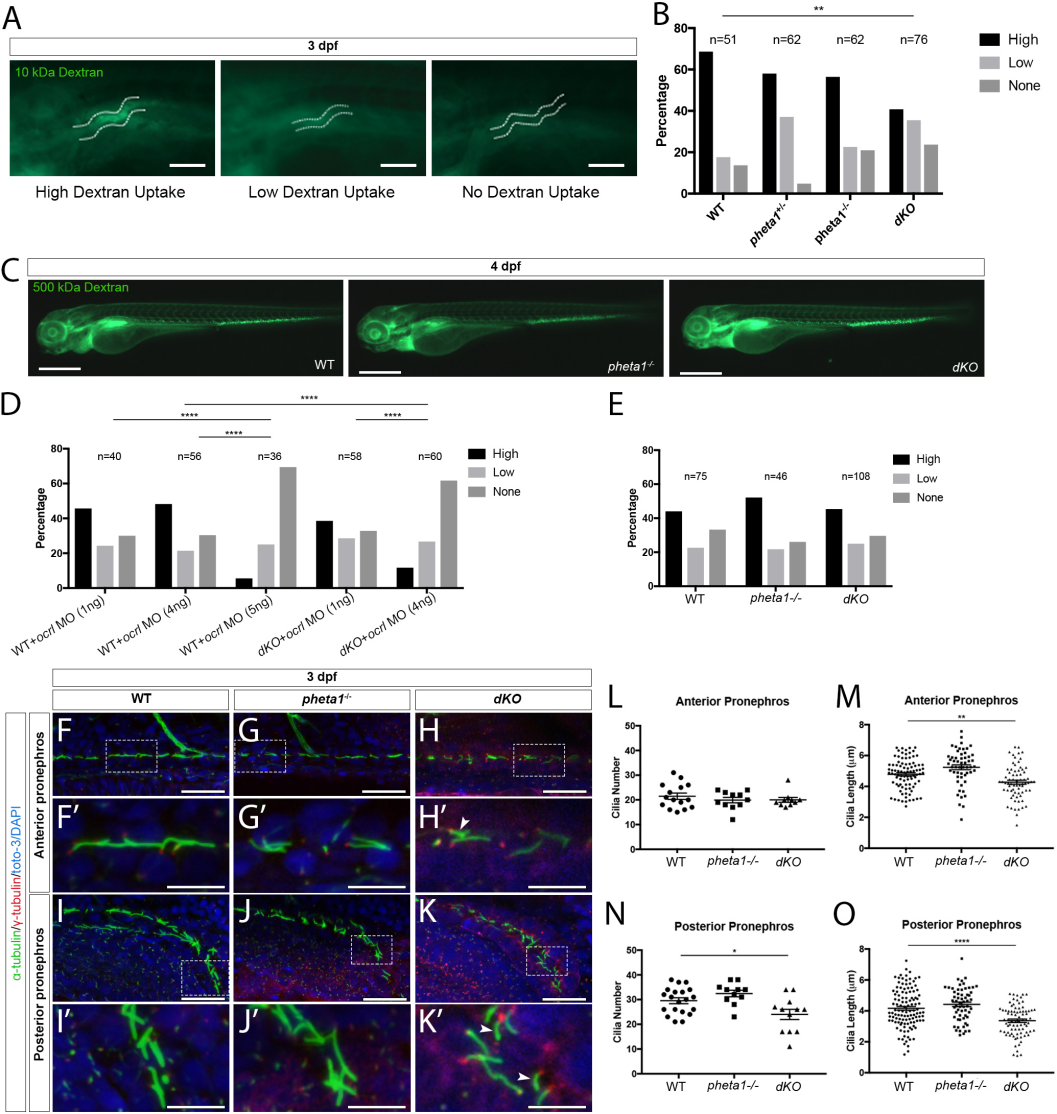
**Loss of *pheta1/2* disrupts fluid-phase endocytosis and ciliogenesis in the pronephros.** (A,B) Pronephros uptake of Alexa Fluor 488-10 kDa dextran in 3 dpf larvae. (A) Animals were categorized as having high, low or no uptake (WT images are shown). The pronephric tubules are indicated by white dashed lines. Scale bars: 100 µm. (B) Comparison of 10 kDa dextran uptake between genotypes. (C) Representative images of animals injected with 500 kDa dextran. Scale bars: 200 µm. (D) WT and *dKO* animals injected with *ocrl* MO at the one-cell stage, then 10 kDa dextran at 3 dpf. (E) Pronephros uptake of RAP-Cy3 at 3 dpf. (F-K′) Representative confocal images of cilia in the pronephros of WT, *pheta1*^−/−^ and *dKO* animals. Cilia were labeled with anti-acetylated α-tubulin (green), basal bodies were labeled with anti-γ tubulin (red), and nuclei were labeled with toto-3 or DAPI (blue). Scale bars: 25 µm. Areas within the white dashed boxes in F,G,H,I,J,K are magnified in F′,G′,H′,I′,J′,K′. Arrowheads indicate examples of shorter cilia in *dKO* animals. Scale bars: 10 µm. (L-O) Quantification of cilia morphology in 3 dpf larvae. Graphs show the cilia number (L) and length (M) in the anterior pronephros, and the cilia number (N) and length (O) in the posterior pronephros. Five cilia were selected from each animal for cilia length measurements. Error=s.e.m. *P<0.05, **P<0.01, ****P<0.0001.

Previous findings in *ocrl*-deficient zebrafish and the UDP patient phenotypes suggested that *pheta1/2* might regulate endocytosis in the renal system (Oltrabella et al., 2015). We examined renal endocytosis in the zebrafish, utilizing an established assay in which fluorescent tracers were injected into the common cardinal vein (CCV), followed by filtration and reabsorption into the renal tubular cells lining the pronephric kidney, commonly referred to as the pronephros (Anzenberger et al., 2006; Drummond et al., 1998; Elmonem et al., 2017; Oltrabella et al., 2015). Endocytic uptake into the renal tubular cells can then be analyzed using fluorescent microscopy. We first tested fluid-phase endocytosis and micropinocytosis, using 10 kDa fluorescent dextran as the tracer (Li et al., 2015). Animals were injected at 3 dpf and then categorized into three groups: high uptake, low uptake or no uptake (Fig. 3A). The *pheta1* heterozygous (*pheta1*^+/−^) and homozygous (*pheta1*^−/−^) animals showed a trend of reduced tracer uptake, compared to the WT control animals, but the difference did not reach statistical significance (Fig. 3B) (Chi-square test; sample sizes are shown in the figure). The *dKO* animals, however, exhibited a significant reduction in tracer uptake compared to WT controls (*P*=0.0085). This suggests that *pheta1* and *pheta2* acted redundantly in fluid-phase endocytosis, such that endocytic deficit was only observed when both proteins were depleted.

To verify that the reduction of 10 kDa dextran uptake in the *dKO* animals was a fluid-phase endocytosis-specific defect, and not due to the disruption of the glomerular filtration barrier, we tested glomerular filtration in the *pheta1^−/−^* and *dKO* animals by injecting 500 kDa dextran. The 500 kDa dextran is too large to pass through a normally functioning glomerular filtration barrier. Therefore, it is expected to remain in the bloodstream. As shown in Fig. 3C, the 500 kDa dextran was retained in the bloodstream in both the *pheta1*^−/−^ and *dKO* animals at 24 h post-injection (hpi), like in the WT controls. Together, these results show that *pheta1/2* are required specifically for fluid-phase endocytosis in the renal organ.

Consistent with the *in vitro* findings that OCRL and PHETA1/2 physically and functionally interact during endosomal trafficking, we found that reduction of zebrafish *ocrl* gene function significantly exacerbated the fluid-phase endocytosis deficits in *dKO* animals. To inhibit *ocrl* function, we used an *ocrl* anti-sense morpholino (MO) that had been validated by *ocrl* mRNA rescue and phenotypic similarity to *ocrl* germline mutants (Coon et al., 2012). To test fluid-phase endocytosis, *ocrl* MO was injected at the one-cell embryonic stage, and then 10 kDa dextran was injected into the CCV at 3 dpf. Sample sizes are as indicated in Fig. 3D. Injection of 5 ng/nl *ocrl* MO in the WT animals resulted in severe reduction in dextran uptake, consistent with the previous finding that *ocrl* deficiency resulted in impaired fluid-phase endocytosis (Oltrabella et al., 2015). Injection of 4 ng/nl *ocrl* MO in WT animals resulted in a partial reduction in dextran uptake. The same *ocrl* MO concentration, however, resulted in a more severe reduction in dextran uptake in *dKO* animals (Chi-square test; *P*<0.0001) (Fig. 3D). This suggests that *ocrl* and *pheta1/2* functionally interact in zebrafish to enable fluid-phase endocytosis. Interestingly, although *ocrl* is required for receptor-mediated uptake of the receptor-associated protein (RAP) (Anzenberger et al., 2006; Oltrabella et al., 2015), we found no significant differences among WT, *pheta1^−/−^* and *dKO* animals in RAP endocytic uptake (Chi-square test; sample size shown in figure) (Fig. 3E). Together, these results indicate that *pheta1/2* are only involved in a subset of the endocytic functions of *ocrl*, specifically fluid-phase endocytosis.

### Loss of *pheta1/2* disrupts ciliogenesis in the pronephros

Several *in vitro* studies have described defects in ciliogenesis after OCRL depletion, and it has been suggested that OCRL regulates protein trafficking to the cilia in a Rab8/PHETA1-dependent manner (Coon et al., 2012; Luo et al., 2012; Rbaibi et al., 2012). To determine if depletion of *pheta1* and/or *pheta2* affect ciliogenesis or cilia maintenance *in vivo*, we analyzed the cilia in the pronephros of 3 dpf larvae (Fig. 3F-K′). We found that *dKO* animals had shorter and fewer cilia, similar to the phenotype seen in *ocrl*-deficient fish (Fig. 3L-O) (Oltrabella et al., 2015). In the *dKO*, cilia length was reduced in the anterior pronephros (one-way ANOVA with Holm-Sidak post-test; WT, *n*=15; *pheta1*^−/−^, *n*=11; *dKO*, *n*=10; *P*=0058) (Fig. 3F-H′), and cilia number and length were both reduced in the posterior pronephros (one-way ANOVA with Holm-Sidak post-test; WT, *n*=20; *pheta1*^−/−^, *n*=11; *dKO*, *n*=12; *P*=0.0213 for cilia number, *P*<0.0001 for cilia length) (Fig. 3I-K′) (Oltrabella et al., 2015). Cilia diameter was not analyzed (due to the resolution limitations of light microscopy). Since *dKO* animals exhibited a similar cilia phenotype to that of the *ocrl* mutants, this further supports the hypothesis that OCRL and PHETA proteins are involved in the same pathway.

We next examined if there was a potential ciliary deficit in other ciliated organs, including the inner ear (macula and crista), the olfactory placode and the lateral line. We found no disruption of ciliogenesis or cilia maintenance in these tissues analyzed in *pheta1^−/−^* and *dKO* animals (Fig. S2). We also examined the outer segments of photoreceptors, which are specialized cilia that originate from the apical-most region of the inner segment. Staining with rod and cone photoreceptor markers did not reveal any differences among WT, *pheta1^−/−^*, *pheta2^−/−^* and *dKO* animals (Fig. S2). This suggests that the role of *pheta1/2* in ciliogenesis is restricted to the pronephros.

### *pheta1/2* is not required for oculomotor function

The UDP patient was born with multiple visual complications, including deficits with oculomotor function (e.g. congenital exotropia, amblyopia, nystagmus). To test whether a deficiency in PHETA1/2 contributes to oculomotor deficits, we analyzed the optokinetic response (OKR) in *pheta1/2* mutants. Zygotic mutants were used to allow for direct comparisons between siblings, which minimized the confounding effects of genetic background-associated variability in behavioral assays (Gorissen et al., 2015; Lange et al., 2013). OKR is a gaze stabilization response that utilizes the extraocular muscles to stabilize an image on the retina in response to visual motion. It is necessary for maintaining optimal visual acuity and is conserved in all vertebrates (Huang and Neuhauss, 2008). In zebrafish, OKR matures at 3-4 dpf but is commonly tested at 5-6 dpf to ensure the complete development of involving neural and extraocular systems (Easter and Nicola, 1997; Huang and Neuhauss, 2008). We tested 5-6 dpf animals inside a circular arena with projections of moving black and white gratings, and the eye positions were video-recorded (Fig. S3A). The grating directions alternated clockwise and counter-clockwise at different contrasts at 3-s periodicity. We tested siblings in the progeny of *pheta1/2* double heterozygous animals (*pheta1^+/–^;pheta2^+/–^*), and the eye velocity in response to the moving gradients was analyzed. No significant differences in eye velocity were observed among WT, *pheta1^−/−^*, *pheta2^−/−^* or *dKO* siblings (two-way ANOVA with Holm-Sidak post-test; WT, *n*=34; *pheta1*^−/−^, *n*=18; *pheta2*^−/−^, *n*=8; *dKO*, *n*=10). We then investigated the correlation of velocity and angle between the left and right eyes (Fig. S3B,C). A reduced correlation would suggest strabismus, in which the eyes do not properly align with each other. No significant differences in angle or velocity correlation were observed among WT, *pheta1^−/−^*, *pheta2^−/−^* or *dKO* siblings (two-way ANOVA with Holm-Sidak post-test; WT, *n*=34; *pheta1*^−/−^, *n*=18; *pheta2*^−/−^, *n*=8; *dKO*, *n*=10). Thus, the deficiency in *pheta1/2* did not affect oculomotor function.

### Loss of *pheta1/2* disrupts craniofacial morphogenesis

**Fig. 4.**
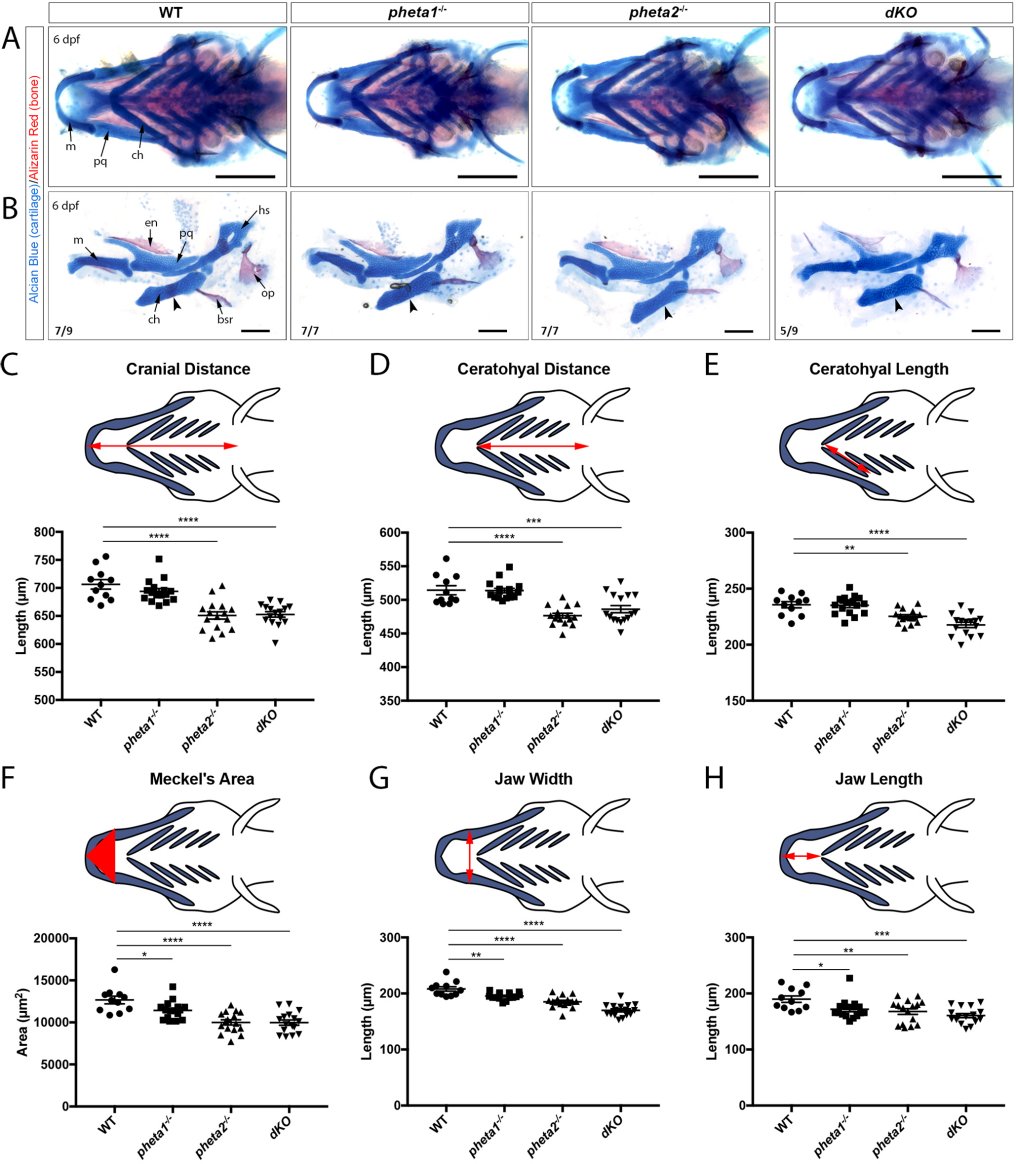
**Loss of *pheta1/2* disrupts craniofacial development.** (A,B) Alcian Blue and Alizarin Red staining in 6 dpf animals. Structures are as indicated in the leftmost images. (A) Ventral view of the lower jaw. Representative images from each genotype are shown. Images are quantified in panels C-H. Scale bars: 200 µm. (B) Flat-mount preparations of the lower jaw at 6 dpf. Arrowheads point to where osteogenesis occurs within the ceratohyal cartilage. The number of animals imaged with displayed phenotype is shown in the lower-left corner of each image. Scale bars: 100 µm. (C-H) Morphological measurements of 6 dpf larvae, with the measured distance/area highlighted in red in the above schematics. bsr, brachiostegal ray; ch, ceratohyal; en, entopterygoids; hs, hyosymplectic; m, Meckel's cartilage; op, opercle; pq, palatoquadrate. *P<0.05, **P<0.01, ***P<0.001, ****P<0.0001.

As mentioned previously, PHETA1 and PHETA2 were previously found to be involved in the sorting of lysosomal hydrolases *in vitro*. Disruption of this pathway *in vivo* could give rise to craniofacial abnormalities, as seen in lysosomal storage disorders such as MLII (Cathey et al., 2010; Koehne et al., 2016; Kudo et al., 2006). Therefore, we investigated whether craniofacial development was affected by the loss of *pheta1/2*. WT, *pheta1^−/−^*, *pheta2^−/−^* and *dKO* animals at 6 dpf were stained with Alcian Blue and Alizarin Red to label the cartilage and bone, respectively. Representative lower jaw images of the WT and mutants are shown (Fig. 4A,B). We did not observe any loss of cartilage or bone structures. There is a reduction in ceratohyal ossification (arrowheads in Fig. 4B), with *pheta2^−/−^* and *dKO* animals lacking the ceratohyal bone collar at 7 dpf (Fig. S4A). This suggests a developmental deficit in the ceratohyal (explored further in the next section).

Overall, the mutants exhibited shorter and narrower jaws. To quantitate this difference, we used a set of morphological parameters previously utilized to describe craniofacial phenotypes in zebrafish larvae, namely cranial distance, ceratohyal distance, ceratohyal length, Meckel's area, jaw width and jaw length (Fig. 4C-H) (de Peralta et al., 2016). Together, these measurements informed us about the overall length and proportions of the head, as well as the growth of an individual cartilage structure (ceratohyal). In the *pheta2^−/−^* and *dKO* animals, we found a significant reduction in all of the above parameters. In the *pheta1^−/−^* animals, only the Meckel's area, jaw width and jaw length were reduced (one-way ANOVA with Holm-Sidak post-test; WT, *n*=11; *pheta1^−/−^*, *n*=16; *pheta2^−/−^*, *n*=16; *dKO*, *n*=16; cranial distance: *P*<0.0001 for WT versus *pheta2^−/−^* and WT versus *dKO*; ceratohyal distance: *P*<0.0001 for WT versus *pheta2^−/−^*, *P*=0.0006 for WT versus *dKO*; ceratohyal length: *P*=0.0066 for WT versus *pheta2^−/−^*, *P*<0.0001 for WT versus *dKO*; Meckel's area: *P*=0.0496 for WT versus *pheta1^−/−^*, *P*<0.0001 for WT versus *pheta2^−/−^*, *P*<0.0001 for WT versus *dKO*; jaw width: *P*=0.0084 for WT versus *pheta1^−/−^*, *P*<0.0001 for WT versus *pheta2^−/−^* and WT versus *dKO*; jaw length: *P*=0.0132 for WT versus *pheta1^−/−^*, *P*=0.0057 for WT versus *pheta2^−/−^*, *P*=0.0003 for WT versus *dKO*) (Fig. 4C-H). Depletion of both *pheta1* and *pheta2* had an additive effect, indicating functional redundancy during craniofacial development.

### Loss of *pheta1/2* disrupts chondrocyte differentiation

**Fig. 5.**
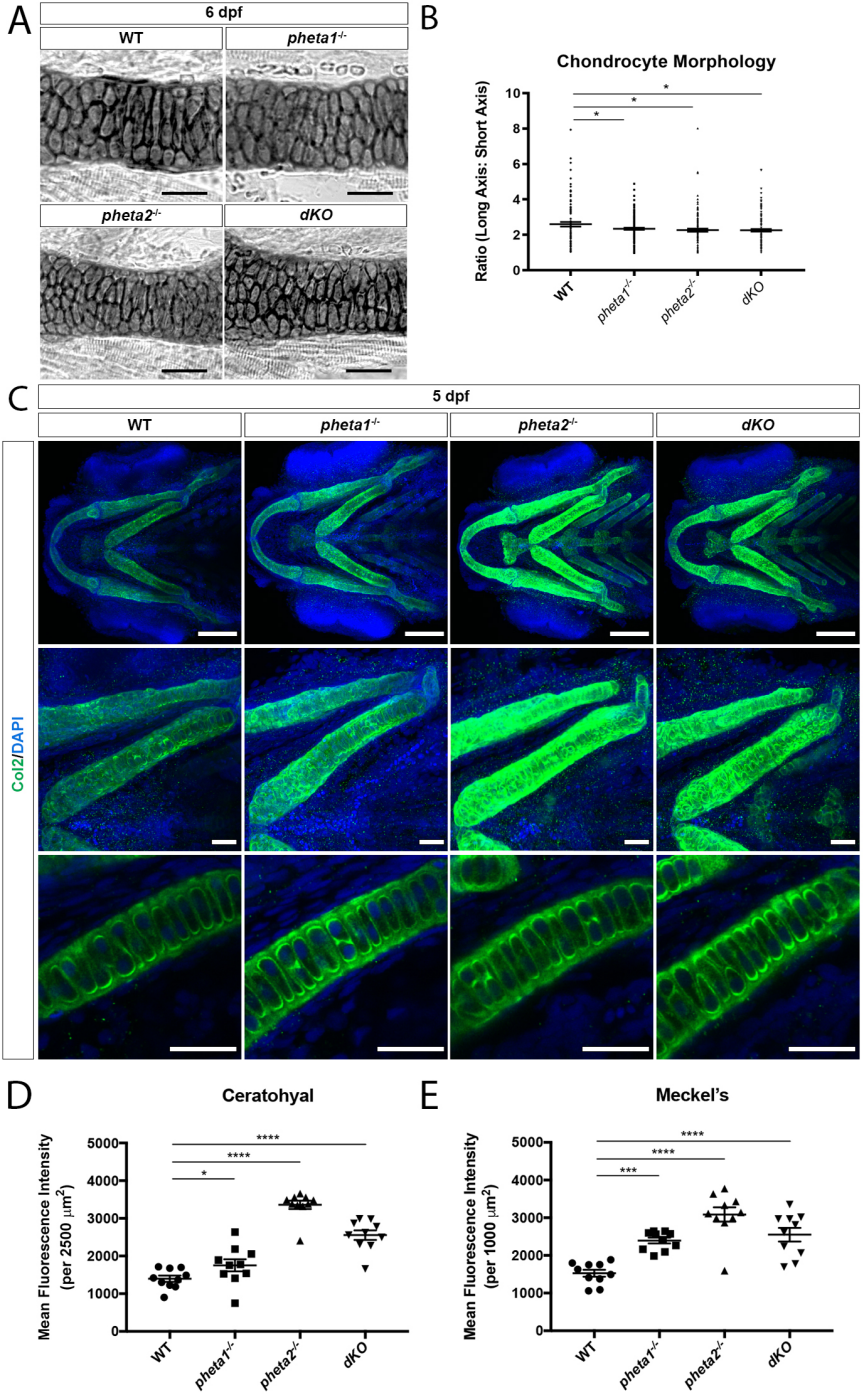
**Loss of *pheta1/2* disrupts chondrocyte maturation.** (A) Ceratohyal cartilage, stained with Alcian Blue in flat-mount preparation. Scale bars: 50 µm. (B) Chondrocyte morphology analysis in ceratohyal cartilage (within a 200 µm^2^ area). (C) Top row: Z-projection ventral view of larvae immunostained for Col2 (green). Nuclei are labeled with DAPI (blue). Scale bars: 100 μm. Middle row: higher-magnification Z-projection images of corresponding ceratohyal cartilage. Scale bars: 25 μm. Bottom row: higher-magnification single optical section images of corresponding ceratohyal cartilage, depicting extracellular secretion of type II collagen. Scale bars: 25 µm. (D,E) Quantification of mean fluorescence intensity in the ceratohyal (D) and Meckel's cartilage (E) in 5 dpf larvae. *P<0.05, ***P<0.001, ****P<0.0001.

The craniofacial morphogenesis defects observed in *pheta1/2* mutants suggest an underlying developmental abnormality. To test this, we first examined the morphology of chondrocytes, which assume a more elongated shape during convergent extension (Kimmel et al., 1998; Le Pabic et al., 2014). If there is a delay in early chondrocyte differentiation, the chondrocytes can persist with more rounded morphology, with a long-axis/short-axis ratio closer to one (Flanagan-Steet et al., 2009; Le Pabic et al., 2014; LeClair et al., 2009). To test this, we measured the long-axis/short-axis ratio of individual cells in flat-mount preparations of the lower jaw (Figs 4B and 5A). One ceratohyal cartilage per animal was imaged, and all cells within the ceratohyal cartilage were measured manually in the Fiji software. We found significant reduction in the elongation of chondrocytes (lower ratio) in the *pheta1^−/−^*, *pheta2^−/−^* and *dKO* animals, suggesting a delay in chondrocyte differentiation (Fig. 5B) (one-way ANOVA with Holm-Sidak post-test; WT, *n*=106 cells from three animals; *pheta1^−/−^*, *n*=137 cells from four animals; *pheta2^−/−^*, *n*=127 cells from three animals; *dKO*, *n*=152 cells from four animals; *P*=0.0439 for WT versus *pheta1^−/−^*, *P*=0.0178 for WT versus *pheta2^−/−^*, *P*=0.0167 for WT versus *dKO*).

Next, we examined the expression of various markers that characterize the sequential stages of development. During chondrocyte differentiation, there is a decline in TGF-β-signaling, and thus a decrease in Smad2/3 and Sox9 transcriptional regulators. At the same time, there is a coordinated change in the extracellular matrix protein composition as the chondrocytes mature (Flanagan-Steet et al., 2016; Goldring et al., 2006). Type II collagen (Col2, encoded by *col2a1a*) is one of the earliest markers of chondrocyte differentiation (Goldring et al., 2006). Col2 expression decreases as chondrocytes become more mature at 4 dpf (Flanagan-Steet et al., 2016). Immunostaining for Col2 at 4 dpf showed that *pheta2^−/−^* and *dKO* animals exhibited a striking increase in Col2 expression compared to WT controls, while the *pheta1^−/−^* animals had a modest increase (Fig. 5C-E) (one-way ANOVA with Holm-Sidak post-test; *n*=10 animals per genotype; ceratohyal: *P*=0.0498 for WT versus *pheta1^−/−^*, *P*<0.0001 for WT versus *pheta2^−/−^* and WT versus *dKO*; Meckel's: *P*=0.0001 for WT versus *pheta1^−/−^*, *P*<0.0001 for WT versus *pheta2^−/−^* and WT versus *dKO*). This is consistent with our morphological measurements, in which *pheta2^−/−^* and *dKO* animals exhibited a more severe deficit in overall craniofacial development compared to *pheta1*^−/−^ animals.

Given the upregulation of Col2, we asked whether other markers of chondrocyte differentiation were affected by the loss of *pheta1/2* (Flanagan-Steet et al., 2016). We examined two later-stage markers, *acana* and *dcn*, which encode the protein cores (Aggrecan and Decorin) of the chondroitin sulfate proteoglycan extracellular matrix. For earlier-stage markers, we looked at *col2a1a* (encodes Col2) and *sox9a* (encodes Sox9). The Sox9 transcription factor is required for early chondrocyte differentiation, but sustained Sox9 expression can inhibit later stages of development (Akiyama et al., 2004; Furumatsu et al., 2005; Yan et al., 2002). Using RNA-seq, WT transcript levels were compared to those from *pheta1^−/−^* and *dKO* (5 dpf). Two-way ANOVA with Holm-Sidak post-test was used for statistical analysis (*n*=3 pools of animals for each genotype). If the *pheta1/2* mutants have more immature chondrocytes, then they would have lower expression of *acana*/*dcn* and higher levels of *col2a1a*/*sox9a.* We found that the expression of *acana* and *dcn* was not significantly changed in the *pheta1^−/−^* and *dKO* groups, compared to WT (Fig. S4B,C). There was a significant increase in *col2a1a* expression in *pheta1^−/−^* and *dKO* groups, consistent with our Col2 immunostaining findings (Fig. S4D) (*P*=0.01 for WT versus *pheta1^−/−^* and WT versus *dKO*). This indicates that increased levels of Col2 may be due to increased *col2a1a* mRNA levels. We found no significant changes in *sox9a* transcript levels (Fig. S4E). *In situ* hybridization also revealed no significant differences in *sox9a* expression in the lower jaw among 4 dpf WT, *pheta1^−/−^*, *pheta2^−/−^* and *dKO* animals (Fig. S4F). Together, we find evidence of delayed chondrocyte differentiation, as evidenced by rounded cell morphology and increased expression of Col2/*col2a1a.* The fact that other markers of chondrocyte differentiation (*acana*, *dcn* and *sox9a*) were unaffected suggests that loss of *pheta1/2* does not globally alter the differentiation program.

### *pheta1/2* regulates Col2 expression through cathepsin K

**Fig. 6.**
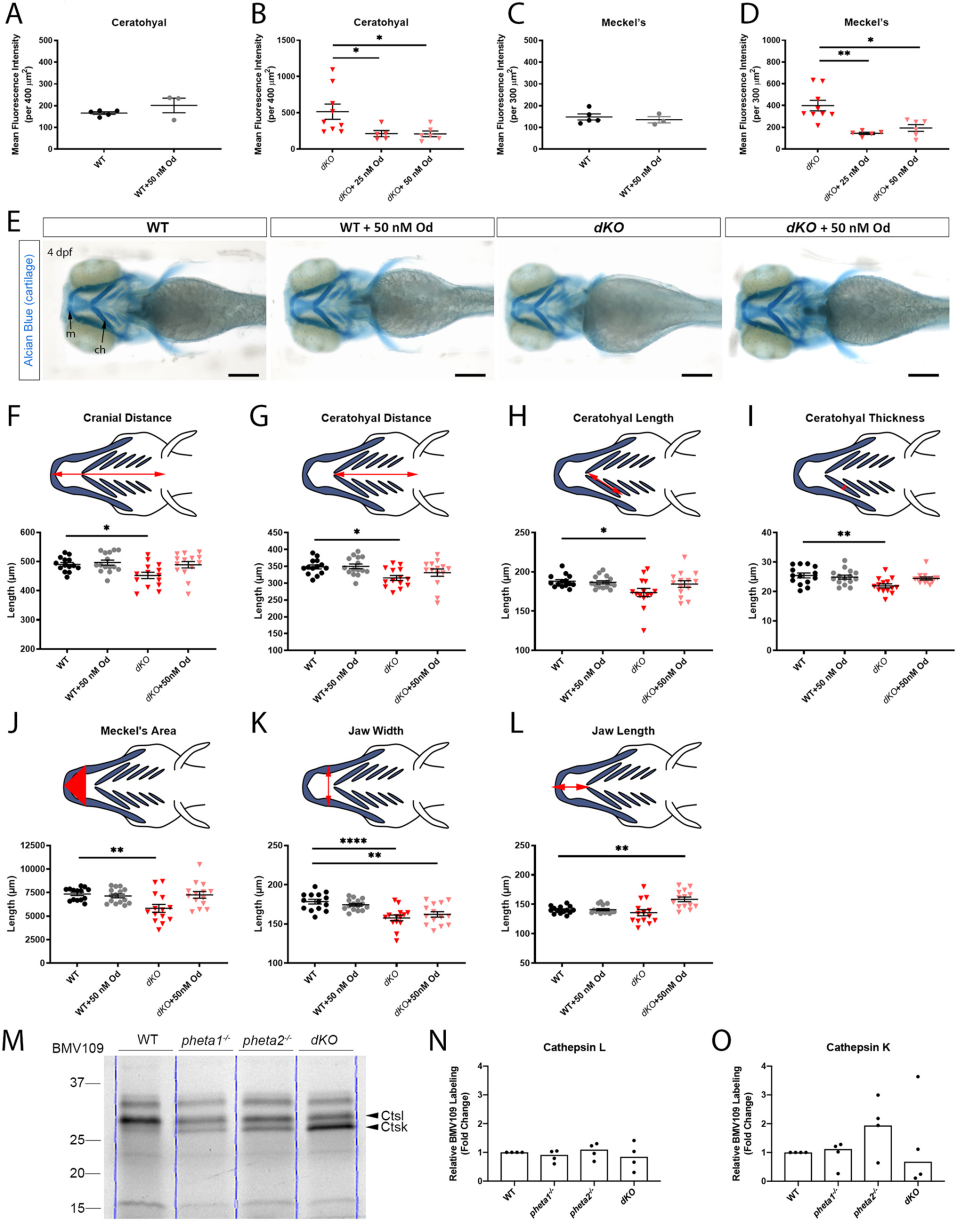
**Craniofacial deficits are rescued by Od-mediated inhibition of cathepsin K.** (A-D) Mean fluorescence intensity of Col2 immunostaining in the ceratohyal (WT in A, *dKO* in B) and Meckel's cartilage (WT in C, *dKO* in D) of 4 dpf larvae with and without Od treatment. (E) Representative images of larvae stained with Alcian Blue. Scale bars: 200 µm. (F-L) Craniofacial morphological measurements at 4 dpf. The measured parameters are highlighted in red in the schematics. (M) In-gel analyses of BMV109, showing cathepsin activities in WT and *pheta1/2* mutants at 4 dpf. Blue lines indicate the lane boundaries. (N,O) Quantitation of the cathepsin K and cathepsin L bands from four experiments. Error=s.e.m. ch, ceratohyal; Ctsk, cathepsin K; Ctsl, cathepsin L; m, Meckel's cartilage. *P<0.05, **P<0.01, ****P<0.0001.

Extracellular matrix remodeling and homeostasis during chondrogenesis and osteogenesis are dependent on the function of proteolytic enzymes, including cysteine proteinases (known as cathepsins) and metalloproteinases (Holmbeck and Szabova, 2006; Yasuda et al., 2005). For example, dysregulation of cathepsin K causes craniofacial abnormalities in zebrafish models of the lysosomal storage disorder MLII (Flanagan-Steet et al., 2018; Petrey et al., 2012). Since PHETA1/2 was found to be involved in the transport of lysosomal hydrolases from the TGN to the endosomes, we hypothesized that cathepsin dysregulation caused by a deficiency in *pheta1/2* might also cause deficits in craniofacial development. To test this, animals were treated with a cathepsin K-specific inhibitor, odanacatib (Od), at 3 dpf and then collected and immunostained for Col2 at 4 dpf (Flanagan-Steet et al., 2018; Gauthier et al., 2008). Od significantly reduced Col2 in the *dKO* animals at both 25 nM and 50 nM concentrations (Kruskal–Wallis test with Dunn's post-test; WT, *n*=5; WT+50 nM Od, *n*=3; *dKO*, *n*=9; *dKO*+25 nM Od, *n*=5; *dKO*+50 nM Od, *n*=5; ceratohyal: adjusted *P*=0.0403 and 0.0323 for 25 nM and 50 nM Od, respectively; Meckel's: adjusted *P*=0.0020 and 0.0176 for 25 nM and 50 nM Od, respectively), while not affecting Col2 levels in WT (Mann–Whitney *U-*test) (Fig. 6A-D). Interestingly, there was no significant Col2 reduction in *pheta1^−/−^* and *pheta2^−/−^*, indicating that *pheta1* and *pheta2* may be able to compensate for one another in regulating cathepsin K activity (Mann–Whitney *U-*test; *pheta1^−/−^*, *n*=6; *pheta1^−/−^*+50 nM Od, *n*=5*^−^*; *pheta2^−/−^*, *n*=5; *pheta2^−/−^*+50 nM Od, *n*=5.) (Fig. S5).

We then tested whether Od could rescue the skeletal phenotypes caused by *pheta1/2* deficiency. To do this, we performed lower-jaw morphological measurements of 4 dpf WT and *dKO* animals with and without exposure to 50 nM Od from 3 dpf to 4 dpf (Fig. 6E). Statistical analyses were performed with one-way ANOVA and Holm-Sidak post-test (WT, *n*=14; WT+50 nM Od, *n*=15; *dKO*, *n*=14; *dKO*+50 nM, *n*=14). Similar to what was found in 6 dpf animals (Fig. 4C-H), *dKO* animals had significantly smaller craniofacial structures, compared to WT (Fig. 6F-L). Specifically, cranial distance (*P*=0.0190), ceratohyal distance (*P*=0.0165), ceratohyal length (*P*=0.0224), ceratohyal thickness (*P*=0.0017) and Meckel's area (*P*=0.0025) were all reduced in *dKO* animals, compared to WT. These metrics were all rescued by Od (*P*>0.05 for WT versus *dKO*+50 nM Od). Jaw width was the only metric not rescued by Od (*P*<0.0001 between WT and *dKO*, *P*=0.0009 between WT and *dKO*+50 nM Od). Interestingly, Od increased jaw length in dKO but not in WT (*P*=0.0021 for WT versus *dKO* treated with Od). The increase in jaw length is not caused by a substantial whole-specimen soft-tissue defect, which was not observed. Together, these results show that the craniofacial deficits in *dKO* animals can largely be rescued by reducing cathepsin K activity with Od.

Next, we asked if *pheta1/2* affects the level of cathepsin K activity. We utilized a cathepsin-specific activity-based probe (ABP), BMV109, to measure global (whole-animal) cysteine cathepsin activity in both WT and *pheta1/2* mutants (Flanagan-Steet et al., 2018; Verdoes et al., 2013). The animals were treated at 3 dpf, a period when certain cathepsin activities (e.g. cathepsin K) typically begin to wane in WT animals (Flanagan-Steet et al., 2018). *pheta1/2* deficiency did not significantly impact cathepsin L activity, the most prominent cathepsin activity at this stage (Kruskal–Wallis test with Dunn's post-test; *n*=5 for all genotypes) (Fig. 6M,N). Cathepsin K activity was also not significantly changed, but there was a trend toward increased activity in *pheta2^−/−^* animals and increased variability in dKO animals (Kruskal–Wallis test with Dunn's post-test; *n*=5 for all genotypes) (Fig. 6M,O). Together, these results indicate that *pheta1/2* may have a specific role in regulating cathepsin K in the cartilage, rather than a global role in cathepsin K activity.

### *pheta1*^R6C^ exerts a dominant-negative effect on craniofacial development

**Fig. 7.**
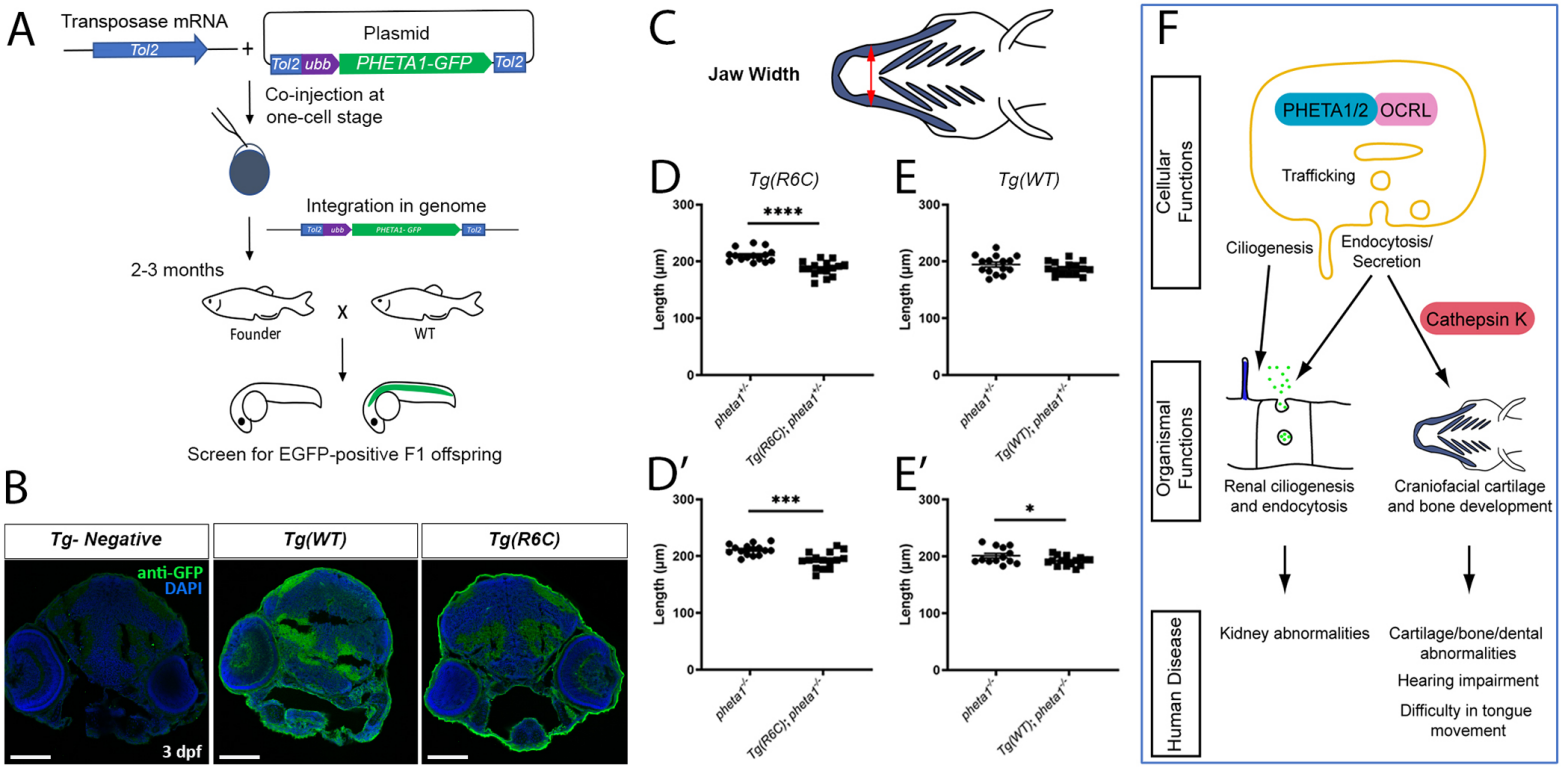
**Pheta1^R6C^ exerts a dominant-negative effect on craniofacial development in the partial or complete absence of Pheta1.** (A) Outline of the procedures for generating the *Tg(R6C)* and *Tg(WT)* transgenic lines. (B) Confocal images showing broad expression of Pheta1^WT^-GFP and Pheta1^R6C^-GFP larvae in transverse cryosections, stained with anti-GFP (green) and DAPI (blue). A control larva with no transgene expression (*Tg-Negative*) is shown for comparison. Scale bars: 100 μm. (C-E′) Craniofacial measurements of 6 dpf larvae, with schematics shown in C. (D,E) Jaw width of *pheta1^+/–^* animals with and without *Tg(R6C)* (D) and *Tg(WT)* (E) transgenes. (D′,E′) Jaw width of *pheta1^−/−^* animals with and without *Tg(R6C)* (D′) and *Tg(WT)* (E′) transgenes. *P<0.05, ***P<0.001, ****P<0.0001. (F) Summary model. At the subcellular level, PHETA1/2 is known to interact with OCRL to regulate intracellular trafficking, ciliogenesis, endocytosis and secretion. These cellular functions likely enable PHETA1/2 to facilitate renal and craniofacial development, with the latter further requiring cathepsin K regulation. In humans, a deficiency in PHETA1 function potentially leads to abnormal development of the kidney and craniofacial structures. Other functional impairments such as hearing and tongue movement may also be associated with abnormal cartilage or bone formation.

One striking clinical feature identified in the UDP patient is abnormal craniofacial development. The patient presented with coarse facial features and facial asymmetry. She also had shorter feet and palms, as well as abnormal dental morphology and malocclusion (Table 1). These phenotypes could be due to cartilage development deficits caused by *PHETA1* haploinsufficiency or dominant-negative effects of the R6C allele. If *pheta1*^R6C^ was non-functional or partly functional, then ectopic expression of Pheta1^R6C^ in *pheta1^+/–^* or *pheta1^−/−^* backgrounds should have no effect or partially improve craniofacial development. However, if *pheta1*^R6C^ was dominant negative, then ectopic expression of *pheta1*^R6C^ should worsen craniofacial development in the same backgrounds. Thus, we generated two zebrafish transgenic lines, one that ubiquitously expressed an EGFP-Pheta1^R6C^ fusion protein [*Tg(ubb:pheta1_R6C-GFP)*, referred to as *Tg(R6C)*], and another that expressed EGFP fused with WT Pheta1 [*Tg(ubb:pheta1-GFP)*, referred to as *Tg(WT)*] (Fig. 7A). Confocal imaging confirmed the broad expression of *Tg(R6C)* and *Tg(WT)* (Fig. 7B).

We then tested whether *Tg(R6C)* and *Tg(WT)* affected craniofacial development. To mimic the genetic background of the UDP patient, which is heterozygous for the R6C allele, we analyzed the effects of *Tg(R6C)* and *Tg(WT)* in the *pheta1* heterozygous (*pheta1*^+/−^) background (Fig. 7C-E). We also analyzed the effects of *Tg(R6C)* and *Tg(WT)* in the *pheta1*^−/−^ background to test whether there could be an effect in the absence of functional *pheta1* (Fig. 7D′,E′). We found that, in both *pheta1^+/–^* and *pheta1^−/−^* backgrounds, *Tg(R6C)* significantly reduced jaw width [two-tailed Student's *t*-test; *pheta1^+/−^*, *n*=15; *Tg(R6C);pheta1^+/−^*, *n*=16; *pheta1^−/−^*, *n*=13; *Tg(R6C);pheta1^−/−^*, *n*=15; *P*<0.0001 for *pheta1^+/–^* background, *P*=0.0004 for *pheta1^−/−^* background]. *Tg(WT)* had no significant effects on jaw width in the *pheta1^+/–^* background [two-tailed Student's *t*-test; *pheta1^+/−^*, *n*=15; *Tg(WT); pheta1^+/−^*, *n*=15; *pheta1^−/−^*, *n*=13; *Tg(WT); pheta1^−/−^*, *n*=15] and a weak negative effect in the *pheta1^−/−^* background (*P*=0.0418). These results indicate that the R6C variant likely acts on craniofacial development in a dominant-negative manner. Interestingly, Pheta1^R6C^ impacted the craniofacial morphological parameters that were affected by the loss of both *pheta1* and *pheta2* (jaw width, Fig. 4G), but not those that were only affected by the loss of *pheta2* (cranial distance and ceratohyal length, Fig. 4C,E; Fig. S6) [two-tailed Student's *t-*test; *pheta1^+/−^*, *n*=15; *Tg(R6C);pheta1^+/−^*, *n*=16; *pheta1^−/−^*, *n*=13; *Tg(R6C);pheta1^−/−^*, *n*=15]. This suggests that Pheta1^R6C^ may have a relatively limited capacity to interfere with Pheta2 function.

## DISCUSSION

The regulation of endocytic trafficking is essential for the development and function of an organism. In this study, we present the first *in vivo* investigation of the functions of PHETA proteins, which are membrane adaptor proteins for the Lowe syndrome causative protein, OCRL. Using zebrafish as the experimental system, we found that *pheta1* and *pheta2* were necessary for renal fluid-phase endocytosis and ciliogenesis. Furthermore, we found that loss of *pheta1/2* impaired craniofacial development and altered the composition of the cartilage extracellular matrix. Evidence also indicates that cathepsin K dysregulation contributes to the craniofacial deficits caused by *pheta1/2* deficiency.

These findings provide insight into the possible pathophysiology of an individual with a *de novo* R6C mutation in PHETA1. The patient presented with renal and craniofacial phenotypes that were similar to the observed phenotypes in *pheta1/2* mutant zebrafish, suggesting that deficiency in PHETA1 contributes to disease. Using transgenic expression in zebrafish, we found that the R6C allele acted in a dominant-negative manner. Together, our results reveal the essential physiological and developmental roles of PHETA proteins and indicate cathepsin proteases as potential targets for PHETA-associated diseases. A summary model is shown in Fig. 7F.

### The roles of *pheta1* and *pheta2* in renal fluid-phase endocytosis and ciliogenesis

Loss of *pheta1/2* affected the renal fluid-phase endocytosis (of 10 kDa dextran substrate), but not receptor-mediated endocytosis (of RAP) (Anzenberger et al., 2006). In contrast, loss of *ocrl* in zebrafish resulted in a strong reduction in both types of endocytosis (Oltrabella et al., 2015). Partial knockdown of *ocrl* in the *dKO* animals exacerbated the fluid-phase endocytic deficit, indicating that *pheta1/2* and OCRL likely function in a common endocytic pathway. These results suggest that *pheta1*/*2* participates in only a subset of the functions of OCRL *in vivo*. Likely, other F&H motif-containing OCRL adaptor proteins such as APPL1 can partially compensate for the loss of PHETA1/2 (Noakes et al., 2011; Pirruccello et al., 2011; Swan et al., 2010).

The pronephros of *dKO* animals had fewer and shorter cilia, similar to what was found in *ocrl*-deficient zebrafish (Oltrabella et al., 2015). However, the ciliogenesis defect in *dKO* and *ocrl^−/−^* animals likely does not account for the endocytosis deficits. First, unabsorbed fluorescent dextran was normally excreted from the cloacae in the *dKO* animals, indicating that there was no impairment of cilia-directed fluid flow within the pronephros. Second, we did not see the development of renal cysts in any of our *pheta1/2* mutants, which is consistent with normal fluid flow. Lastly, even mutants with severe ciliogenesis deficits (e.g. the *double bubble* mutant) could endocytose dextran normally (Drummond et al., 1998; Liu et al., 2007; Oltrabella et al., 2015). Thus, *pheta1/2* likely contributes to fluid-phase endocytosis independently of its role in ciliogenesis.

### A novel role for *pheta1* and *pheta2* in craniofacial development

We identified a novel role for *pheta1* and *pheta2* in craniofacial morphogenesis. Craniofacial development appeared to rely more on *pheta2*, but depletion of both *pheta1* and *pheta2* resulted in an additive effect, indicating that *pheta1* plays a role as well. In *pheta2^−/−^* and *dKO* animals, we observed features indicative of abnormal chondrocyte differentiation, including abnormal chondrocyte morphology, reduced ceratohyal ossification, changes in marker gene expression and altered extracellular matrix composition (i.e. increased Col2). As a first foray into the underlying molecular mechanisms, we found that inhibition of cathepsin K using the specific inhibitor Od significantly reduced Col2 protein levels in the *dKO* animals and rescued most of the lower jaw morphological deficits in the *dKO* animals. This indicates that overactive cathepsin K activity may be the cause of abnormal craniofacial development.

Interestingly, we did not see a consistent global increase in cathepsin K activity using an *in vivo* activity probe (BMV109), indicating that the dysregulation of cathepsin K activity may stem from changes in a subset of cells within craniofacial structures. Alternatively, there may be clutch-to-clutch differences in compensatory mechanisms to control cathepsin K activity. For example, there may be variability in cystatin activity, which inhibits cathepsin K (Vidak et al., 2019). Cathepsins also regulate growth factor activity in the cellular microenvironment, which may result in stochastic changes and more variability during the course of development. Future studies might explore where active cathepsin K resides as development progresses and how Col2 levels are modulated by protease activity in *pheta1/2* mutant animals. It was previously shown that TGF-β signaling is enhanced by mislocalized cathepsin K activity (Flanagan-Steet et al., 2016, 2018; Vidak et al., 2019). Thus, the absence of *pheta1/2* could lead to altered TGF-β signaling, which may, in turn, mediate the abnormal craniofacial morphogenesis.

### Investigating the pathogenesis of the UDP patient's disease

A primary motivation for understanding the *in vivo* function of PHETA1/2 was the identification of a patient carrying a *de novo* PHETA1 mutation. To the best of our knowledge, this patient was the first reported case of human disease associated with PHETA1 or PHETA2 mutation. Although the R6C mutation did not affect interaction with OCRL, it did exert a dominant-negative effect on craniofacial development, even in the absence of endogenous *pheta1*. Since the R6C mutant can interact with OCRL, it may be able to disrupt the function of OCRL complexes, analogous to how the G59S mutation in dynactin subunit 1 (DCTN1) disrupts the function of the dynein/dynactin complex (Lai et al., 2007). Alternatively, since PHETA1 and PHETA2 can form homodimers and heterodimers, the R6C mutant may bind to and interfere with the normal functions of PHETA1 and PHETA2. Our hypothesis that the R6C mutation resulted in a deficiency of PHETA1/2 function is supported by the overlapping phenotypes between the patient and our zebrafish mutants, specifically in craniofacial development and renal function (Table 1).

When drawing comparisons between zebrafish and human craniofacial phenotypes, it is important to note the relationships between zebrafish lower-jaw elements and human jaw anatomy (DeLaurier, 2019; Mork and Crump, 2015). The first pharyngeal arch (Meckel's, palatoquadrate)- and second pharyngeal arch (hyosymplectic, ceratohyal)-derived elements become the lower jaw and craniofacial skeleton in both species. The Meckel's cartilage gives rise to part of the lower jaw and the inner ear, whereas the ceratohyal gives rise to the styloid process and the hyoid. With this in mind, the deficits we observed in Meckel's and ceratohyal cartilage could provide plausible explanations to some of the UDP patient's clinical presentations. Specifically, dental abnormalities and hearing impairments may be linked to deficits in the Meckel's cartilage, whereas difficulty in tongue movements could be caused by deficits in the hyoid, which is a ceratohyal-derived structure (Fig. 7F).

Lastly, we note that the UDP patient has three other *de novo* mutations considered less likely to be contributing to disease. One variant in DnaJ heat shock protein family (Hsp40) member B5 (*DNAJB5*; NM_001135004: p.R419H) has inconsistent predictions with SIFT and Polyphen, and occurs in a moderately conserved amino acid, so it is unlikely that this causes the UDP patient's disease. A second variant, in uridine phosphorylase 1 (*UPP1*; NM_003364:p.I117V), is seen in 12 normal individuals and is predicted benign by SIFT and Polyphen, so it is unlikely to be pathogenic. The third variant, is in plant homeodomain (PHD)-like finger protein 6 (*PHF6*; NM_001015877.1: p.Leu244del), which has been associated with X-linked Borjeson–Forssman–Lehmann syndrome (BFLS; MIM #301900); one female patient has been reported with a loss of function allele and X-inactivation (Turner et al., 2004). X-inactivation studies in our patient showed a skewed pattern, but an association with PHF6 was unlikely due to a lack of phenotypic overlap with BFLS. Furthermore, the variant identified in our patient, unlike a clear loss of function mutation reported in BFLS, leads to an in-frame deletion with no splicing defect (Fig. S7). Identification of additional patients carrying deleterious PHETA1 mutation will help to clarify which phenotypes are more closely associated with PHETA1 deficiency in humans.

## Conclusions

In conclusion, we have determined novel *in vivo* functions of the OCRL adaptor proteins PHETA1 and PHETA2. Deficiency in *pheta1/2* resulted in impaired renal physiology and craniofacial development in zebrafish, resembling the renal and craniofacial phenotypes in a UDP patient carrying a dominant-negative allele of PHETA1. The craniofacial deficits in zebrafish *pheta1/2* mutants were likely caused by a dysregulation of cathepsin K, which altered the extracellular composition of craniofacial cartilages and craniofacial morphogenesis. These results support the hypothesis that PHETA1 mutation was contributory to disease, but further studies with additional patients will be needed to determine the roles of PHETA1/2 in human disease fully.

## MATERIALS AND METHODS

### Patient enrollment, consent and sample analysis

The patient (UDP.5532) was enrolled in the NIH UDP (Gahl et al., 2012, 2016, 2015) under the protocol 76-HG-0238, ‘Diagnosis and Treatment of Patients with Inborn Errors of Metabolism and Other Genetic Disorders’, which was approved by the Institutional Review Board of the National Human Genome Research Institute. Written informed consent to publish was obtained from the parents of the patient.

Patient-derived fibroblasts were cultured in high-glucose Dulbecco's modified Eagle medium (DMEM) supplemented with 15% fetal bovine serum (FBS), non-essential amino acid solution, and penicillin-streptomycin with L-glutamine (Thermo Fisher Scientific). Normal adult human sex-matched dermal fibroblasts (ATCC PCS-201-012) were used as controls. Cell cultures were checked regularly for contamination. RNA was isolated using an RNeasy Mini Kit (Qiagen), and first-strand cDNA was synthesized by a high-capacity RNA to cDNA kit (Thermo Fisher Scientific) according to the manufacturer's protocol. For qRT-PCR, primer pairs specific to the three common isoforms (NM_001177996.1, NM_001177997.1 and NM_144671.4) of human *PHETA1* (forward primer, 5′-GAAGAGCGAGCTGAGGCTG-3′; reverse primer, 5′-GTCACAGGTGGCGTAGAAGG-3′) and housekeeping gene *POLR2A* (forward primer, 5′-CATGTGCAGGAAACATGACA-3′; reverse primer, 5′-GCAGAAGAAGCAGACACAGC-3′) were PCR amplified and monitored using a CFX96 Touch Real-Time PCR detection system (Bio-Rad). Relative expression of *PHETA1* transcripts was normalized to the expression of *POLR2A* and analyzed using standard delta delta Ct method. qRT-PCR experiments were performed in accordance with the Minimum Information for Publication of Quantitative Real-Time PCR Experiments (MIQE) guidelines (Bustin et al., 2009). For splice site analysis of the variant in *PHF6* (NM_001015877.1:c.732_734del; p.Leu244del), we amplified the patient cDNA using *PHF6*-specific primers flanking the site of mutation and subcloned into a plasmid vector using TOPO-TA cloning (Thermo Fisher Scientific), and sequenced according to the manufacturer's instructions. Recombinant colonies were picked up by blue-white screening and extracted plasmids were sequenced using vector-specific M13 primers.

### Zebrafish husbandry

Zebrafish of both sexes and all ages were maintained under a standard protocol in accordance with Institutional Animal Care and Use Committee guidelines at Augusta University, Virginia Tech and Greenwood Genetic Center. All zebrafish used in this study were in a mixed background of AB and TL WT lines (Zebrafish International Resource Center). Sex is not a relevant variable for the stages being used (0-7 dpf), as laboratory zebrafish remain sexually undifferentiated until 2 weeks of age (Maack and Segner, 2003; Wilson et al., 2014). To prevent pigment formation for selected experiments, embryos were transferred to embryo medium containing 0.003% 1-phenyl-2-thiourea (PTU; Sigma-Aldrich) between 18 h post-fertilization (hpf) and 24 hpf.

### Mutant and transgenic zebrafish lines

*pheta1* (*si:ch211-193c2.2*, ZFIN ID: ZDB-GENE-041210-163, Chromosome 5: 9,677,305-9,678,075) was identified by a BLAT search using a human *PHETA1* coding sequence against the UCSC zebrafish genome database (Kent, 2002). *pheta2* (*zgc:153733*, ZFIN ID: ZDB-GENE-060825-273, Chromosome 3: 32,821,205-32,831,971) was identified as a paralog of *pheta1* in the Ensembl database (Zerbino et al., 2018). Neighboring genes of *pheta1/2* were identified using the UCSC genome browser (Kent et al., 2002). Phylogenetic tree of PHETA proteins was inferred using the Neighbor-Joining method in the MEGA X software (Kumar et al., 2018). The evolutionary distances were computed using the Poisson correction method. An *in silico* search for *pheta1/2* paralogs was performed, utilizing BLAST and the Comparative Genomics tool in the Ensemble website (ensemble.org). No *pheta1/2* paralogs were identified. A search was performed on the UCSC genome database for potential syntenic regions between human and zebrafish. In humans, *PHETA1* is adjacent to *CUX2*, *SH2B3* and *ATXN2*, whereas *PHETA2* is adjacent to *NAGA*, *SMDT1* and *NDUFA6*. The genomic regions containing the zebrafish homologs of these genes did not contain any additional PH domain-containing genes in these regions. These findings support the idea that *pheta1/2* are the only zebrafish homologs of human *PHETA1/2*. Protein sequence similarity was calculated using the AlignX module from the Vector NTI 9 software suite (Invitrogen).

Mutants were generated using CRISPR-Cas9 genome engineering, as previously described (*pheta1* target sequence, GGAGCTGAACGAGAGGAGTG; *pheta2* target sequence, GGTCTCTGACTATCATGGAG) (Gagnon et al., 2014; Montague et al., 2014). The *pheta1*^*vt2*^ allele harbored a 38 bp deletion (frameshift), resulting in the deletion of a MwoI restriction site, which was used to distinguish between WT and *pheta1*^*vt2*^ alleles. Genomic DNA flanking the deletion was amplified by PCR, followed by MwoI digestion for 2 h at 60°C (primer sequences 5′-CCTCAAACAAACTAGCGGACGTGTCGAGTA-3′ and 5′-CGCGACAGAGCCTTTACCCATGATTCCATA-3′). After MwoI digestion, the cut WT bands were 230 bp and 300 bp in length, whereas the mutant band was 531 bp (uncut). The *pheta2^vt3^* allele harbored an 11 bp deletion, resulting in the deletion of an NlaIII restriction site, which was used to distinguish between WT and *pheta2*^*vt3*^ alleles. Genomic DNA flanking the deletion was amplified by PCR, followed by NlaIII digestion for 2 h at 37°C (primer sequences 5′-GGACGGTCAGTTCTGTTTCTCT-3′ and 5′-CATGTAAACATACCTTCGTATCGTC-3′). After NlaIII digestion, the cut WT bands were 180 bp and 44 bp in length, whereas the mutant band was 213 bp (uncut).

*Tg(ubb:pheta1-GFP)vt4* and *Tg(ubb:pheta1_R6C-GFP)vt5* transgenic zebrafish lines were generated utilizing the Tol2-transgenesis system (Kawakami, 2007). Coding sequence for EGFP was ligated in frame to the 3′ end of the coding sequence of either the WT (Pheta1-GFP) or the patient-specific (Pheta1_R6C-GFP) Pheta1 protein, and placed into the Tol2 vector, preceded by the zebrafish *ubiquitin* promoter from Mosimann et al. (2011). The *Tol2-ubb:pheta1-GFP* and *Tol2-ubb:pheta1_R6C-GFP* vectors were then injected with Tol2 transposase mRNA into WT zebrafish larvae at the one-cell stage. Potential founders were crossed to WT fish at 2-3 months of age, and offspring were screened for EGFP-positive F1 founders.

### RT-PCR analysis of *pheta1* and *pheta2* transcript

Total RNA was isolated from pools of animals using an RNA Miniprep Kit (Zymo). To determine whether CRISPR-induced deletions were incorporated into transcribed mRNA, 50 ng of total RNA from 4 dpf WT, *pheta1^−/−^* and *pheta2^−/−^* animals were used for first-strand cDNA synthesis (SuperScript III, Thermo Fisher Scientific), followed by PCR amplification (GoTaq G2 Green Master Mix, Promega). Purified PCR products were sequenced by Sanger sequencing at the Virginia Tech Genome Sequencing Center. Sequencing results confirmed that the CRISPR-induced 38 bp and 11 bp deletions were incorporated into the *pheta1* and *pheta2* transcripts, respectively. To determine the expression of *pheta1* and *pheta2* during early development, 300 ng of total RNA from 512-cell, 1 dpf, and 3 dpf WT zebrafish was used for first-strand cDNA synthesis (LunaScript RT SuperMix, NEB), followed by PCR amplification. Primers used were as follows: *pheta1*, 5′-GGAAGAATCAAGGGAGAAAAACTGCG-3′ and 5′-TCCTCGAAGTAGAACAGCATGTTGCC-3′; *pheta2*, 5′-ACCCATTACCTGTCCTGCACTTCAC-3′ and 5′-CTAGCCAAGATCAATGAGGTCCTCCTC-3′; *rpl4*, 5′-GTGCCCGACCGTTAATCTC-3′ and 5′-ACACTGCTGGCATAACCACAT-3′.

### Whole-mount *in situ* hybridization

*In situ* hybridization was performed using protocols described previously (Pan et al., 2012; Prober et al., 2008). Sense and antisense probes were transcribed from linearized plasmid DNA using a DIG RNA Labeling Kit (Roche). *pheta1 in situ* probes were synthesized using *pheta1*-5′UTR sequence (primer sequences 5′-TGGATCCGGAAGAATCAAGGGAG-3′ and 5′-TCTCGAGGAACAGCATGTTGCC-3′). The *sox9a* anti-sense probes have been described previously (Flanagan-Steet et al., 2016).

### Histochemistry and immunohistochemistry

Alcian Blue/Alizarin Red staining was performed using the ‘No acid’ protocol (Walker and Kimmel, 2007). Briefly, after fixation with 2% paraformaldehyde and rinse with 50% ethanol, samples were stained overnight in 0.04% Alcian Blue (Anatech)/0.01% Alizarin Red S (Sigma-Aldrich)/10 mM MgCl_2_/80% ethanol. Stained samples were rinsed in 80% ethanol/10 mM MgCl_2_ for several hours, and washed in 50% and 25% ethanol. After washing, samples were bleached in 3% H_2_O_2_/0.5% KOH for 10 min with the cap open, followed by rinsing in 25% and 50% glycerol/0.1% KOH, and stored in 50% glyceron/0.1% KOH. For Od treatment experiments, Alizarin Red S was not included in the staining solution. Flat mount preparation was performed as described by Javidan and Schilling (2004). Live staining of ceratohyal bone collar was performed as described previously (Flanagan-Steet et al., 2016). Fish at 7 dpf were placed in Eppendorf tubes and stained in 0.05% Alizarin Red/10 mM HEPES pH 7.0 (Fisher Scientific)/E3 for 1 h in the dark, rinsed in 10 mM/10 mM HEPES pH 7.0, and anesthetized in 0.013% tricaine (Fisher Scientific)/E3. Anesthetized animals were mounted face down on an uncoated 50 mm glass-bottom Petri dish (MatTek) in 1.7% low melting agarose (Fisher Scientific) in E3 buffer for imaging.

Immunohistochemistry was performed as previously described (Randlett et al., 2015). Primary antibodies were as follows: anti-acetylated α-tubulin (T6793; Sigma-Aldrich; 1:1000), anti-γ-tubulin (T5326; Sigma-Aldrich; 1:100), anti-Znp-1 [ANZNP-1; Developmental Studies Hybridoma Bank (DSHB); 1:25], anti-Zpr1 [zpr1; Zebrafish International Resource Center (ZIRC); 1:100], anti-Zpr3 (zpr3; ZIRC; 1:100), anti-Collagen type II (II-II6B3; DHSB; 1:100), anti-GFP (ab13970; Abcam; 1:1000). Alexa Fluor-conjugated secondary antibodies (Life Technologies; 1:500), 4′,6-diamidino-2-phenylindole (DAPI; D1306; Life Technologies; 1:1000) and toto-3 (T3604, Life Technologies, 1:2000) were used after primary antibody incubation.

To image Alcian Blue/Alizarin Red-stained samples, individual fish were mounted on a glass slide in 50% glyceron/0.1% KOH and imaged using a Nikon SMZ18 fluorescent stereomicroscope with an image capture system. To image ceratohyal chondrocytes, flat-mount specimens were imaged under bright-field illumination using a Zeiss Axio Imager 1 upright compound microscope with a 20× objective. For fluorescent imaging, animals were mounted on an uncoated 50 mm glass-bottom Petri dish (MatTek) in 1.7% low melting agarose (Fisher Scientific) and imaged with a Nikon SMZ18 fluorescent stereomicroscope or a Nikon A1 laser scanning confocal system with a CF175 Apochromat LWD 25× water-immersion objective. The same imaging settings were used for all samples in each experiment.

### Injection of endocytic tracers and analysis

Lysine-fixable 10 kDa dextran (Alexa Fluor 488 conjugated) or 500 kDa dextran [fluorescein isothiocyanate (FITC) conjugated] (Thermo Fisher Scientific) were prepared in PBS at 2 µg/µl final concentration. In addition, recombinant Cy3-conjugated His-tagged RAP (39 kDa), prepared in PBS at 5 µg/µl final concentration, was kindly provided by Dr Martin Lowe (University of Manchester, Manchester, UK). Zebrafish embryos were anesthetized in tricaine (0.013% w/v; Fisher Scientific) diluted in embryo water at 72 hpf. Approximately 0.5-1 nl of dextran or RAP was injected into the common cardinal vein using a glass micropipette and a pneumatic pressure injector (PLI90; Harvard Apparatus) and micromanipulator. Uptake in the renal tubular cells of the proximal pronephros was analyzed at 1-2.5 hpi, using a Nikon SMZ18 fluorescent stereomicroscope with an image capture system. High dextran uptake was defined as >20 fluorescent puncta observed along the proximal pronephros. Low dextran uptake was defined as one to 20 fluorescent puncta observed along the proximal pronephros, and no uptake indicated that no fluorescent puncta were seen. Animals injected with 500 kDa dextran were analyzed at 24 hpi.

### MO inhibition of *ocrl* gene expression

The *ocrl* translation-blocking MO (a gift from Dr Martin Lowe, University of Manchester) has previously been described (sequence AATCCCAAATGAAGGTTCCATCATG) (Coon et al., 2012). MO was injected into embryos at the one-cell stage at 1-5 ng/µl.

### Cilia and craniofacial quantification and analysis

Cilia in the anterior (just anterior to the yolk extension) and posterior (near the cloacae) portion of the pronephros in the zebrafish larvae were imaged and analyzed. The number of cilia within a 100×100 µm^2^ area were quantified, and the length of five randomly selected cilia was measured within the area. Craniofacial morphological measurements were performed with Fiji (Schindelin et al., 2012). Type II collagen was quantified by mean fluorescence intensity within a 2500 µm^2^ area in the ceratohyal cartilage and a 1000 µm^2^ area in Meckel's cartilage.

### BMV109 delivery and in-gel analyses

The BMV109 fluorescent ABP was injected into 3 dpf larvae (1 nl at 10 µM) pericardially via microinjection. This equates to a final global concentration of 10 nM. Probe was circulated overnight at 28.8°C and harvested at 15 hpi. Twenty-five larvae per condition were collected and lysed in citrate buffer (50 mM citrate buffer pH 5.5, 5 mM DTT, 0.5% CHAPS, 0.75% Triton X-100) by brief sonication. Samples were centrifuged for 15 min at 15,000 ***g*** and the supernatant collected. Protein concentration was determined via a micro BCA assay (23235; Thermo Fisher Scientific) and samples run on 4-20% precast gradient gels containing the ‘stain free’ tri-halo compound (Bio-Rad). UV light-activated tri-halo covalently binds tryptophan residues. Equivalent protein loads were evaluated on a Bio-Rad Chemidoc MP Imaging System using this stain-free method. BMV109 Cy5 fluorescence was subsequently analyzed in gel. Total protein load per lane and individual ABP-reactive bands were quantitated using Chemidoc MP software. Individual ABP-reactive bands were normalized to total protein load and the fold difference calculated between WT and MLII samples.

### Pharmacological inhibition

Cathepsin K activity was inhibited from 3 dpf to 4 dpf in live embryos by introducing 25 nM and 50 nM Od [solubilized in dimethyl sulfoxide (DMSO)] directly into their growth medium. In all cases, WT control larvae were treated with an equivalent amount of DMSO (0.1%).

### Cell culture

HeLa cells (ATCC CCL-2) were grown in DMEM supplemented with 5% FBS (Thermo Fisher Scientific) and 1% penicillin-streptomycin (Life Technologies). Cells were transfected using Effectene (Qiagen) according to instructions provided by the manufacturer. The *PHETA1* cDNA was synthesized by Genewiz and cloned into the pEGFP-C3 vector (Promega). The R6C mutation was introduced into the construct using a Q5 site-directed mutagenesis kit (NEB). The pcDNA3-HA-human OCRL plasmid was Addgene plasmid #22207 (http://n2t.net/addgene:22207; RRID:Addgene_22207), deposited by Pietro De Camilli.

### Protein-protein interaction

Lysates were prepared from transfected HeLa cells by incubating the cell pellet in RIPA buffer (50 mM Tris-Cl pH 7.5, 150 nM NaCl, 1% NP40, 1 mM EDTA). The lysate was clarified by centrifugation at 10,000 ***g*** for 5 min at 40°C. Then, 1× Halt protease inhibitor cocktail (Pierce) was added to the lysate, with 800 µg total protein used per immunoprecipitation. Immunoprecipitation was performed using GFP-Trap beads (Chromotek) in binding buffer (50 mM Tris pH 7.5, 150 mM NaCl, 0.2 mM EDTA, 0.05% NP40). The lysate was incubated with beads for 75 min at 40°C. Subsequently, the beads were washed four times using binding buffer. The bound proteins were eluted by boiling in Laemmli buffer, run on a gel and analyzed by western blotting. A monoclonal GFP antibody (JL-8; Clontech) and a monoclonal hemagglutinin (HA) antibody (sc-7392; Santa Cruz Biotechnology) were used in the western blot analyses. The HRP signal was acquired on a Chemidoc MP (Bio-Rad) imaging system.

### RNA-seq analysis of transcript abundance in zebrafish

Total RNA from WT, *pheta1^−/−^* and dKO larvae (5 dpf, head only) was isolated using an RNA Miniprep Kit (Zymo). Three biological replicates for each group were analyzed, each containing RNA from a pool of ten animals. All samples had an RNA integrity number ≥8.0 and were converted into a strand-specific library using Illumina's TruSeq Stranded mRNA HT Sample Prep Kit (Illumina, RS-122-2103) for subsequent cluster generation and sequencing on Illumina's NextSeq. The library was enriched by 13 cycles of PCR, validated using Agilent TapeStation and quantitated by qPCR. Individually indexed cDNA libraries were pooled and sequenced on NextSeq 75 SR to obtain a minimum of 30 million reads/sample. Following sequencing, data were trimmed for both adaptor and quality using a combination of ea-utils and Btrim (Aronesty, 2013; Kong, 2011). Sequencing reads were then aligned to the genome (Ensembl Danio_rerio.GRCz11.92 with and without unplaced contigs) using Tophat2/HiSat2 (Kim et al., 2015) and counted via HTSeq (Anders et al., 2015). Read counts for genes annotated on the unplaced contigs were added to the chromosome-only count summary. QC summary statistics were examined to identify any problematic samples [e.g. total read counts, quality and base composition profiles (+/− trimming)], raw fastq-formatted data files, aligned files (bam and text file containing sample alignment statistics) and count files (HTSeq text files). The data discussed in this publication have been deposited in NCBI's Gene Expression Omnibus (GEO) (Edgar et al., 2002) and are accessible through GEO series accession number GSE142673 (https://www.ncbi.nlm.nih.gov/geo/query/acc.cgi?acc=GSE142673). The expression of *pheta1*, *pheta2* and condrogenesis genes (*col2a1a*, *acana*, *dcn*, *sox9a*) was compared using two-way repeated-measures ANOVA with Holm-Sidak correction.

### OKR in zebrafish

VisioTracker 302060 (New Behavior TSE) was used for OKR assay. Eye movements of individual fish were recorded at five frames/s by an overhead CCD camera. Zebrafish larvae were placed in the center of an uncoated 50 mm glass-bottom Petri dish (MatTek) and immobilized in 1.5-2% low melting agarose (Fisher Scientific) in E3 buffer. Agarose around the eye was removed with forceps to allow free eye movement. The dish was then filled with water. To test slow-phase performance under short periodicity, the direction of black and white grating switched every 3 s with grating velocity at 7.5°/s. Each experimental run (trial) was 108 s long and included twelve 9-s phases at varying contrast levels (0.99, 1.0, 0.5, 0.2, 0.1, 0.05, 0.02, 0.05, 0.1, 0.2, 0.5, 1.0). Five to six trials were tested for each animal. Contrast sensitivity and eye correlation were calculated using custom MATLAB scripts (available upon request).

### Image processing and statistical analyses

Images were quantified with Fiji (Schindelin et al., 2012), and figures were made in Photoshop (Adobe Systems). All statistical analyses were performed in GraphPad Prism (Version 8). Chi-square tests were used to analyze categorical data from endocytosis assays. For normally distributed data, parametric tests (Student's *t-*test or ANOVA) were used. For ANOVA tests, the Holm-Sidak post-test was performed to correct for multiple comparisons. For non-normally distributed data, non-parametric tests (Mann–Whitney *U*, Kruskal–Wallis) were used. Dunn's correction was used to correct for multiple comparisons after Kruskal–Wallis tests. All values are expressed as mean±s.e.m., unless otherwise noted. The test was considered significant when *P*<0.05.

## Supplementary Material

Supplementary information
