# Supplementary material for: Deficiency in the endocytic adaptor proteins PHETA1/2 impairs renal and craniofacial development
Source: Dis Model Mech. 2020 May 26;13(5):dmm041913. doi: 10.1242/dmm.041913 (PMC7272357; doi:10.1242/dmm.041913)
Supplement: Supplementary information [file dmm-13-041913-s1.pdf]

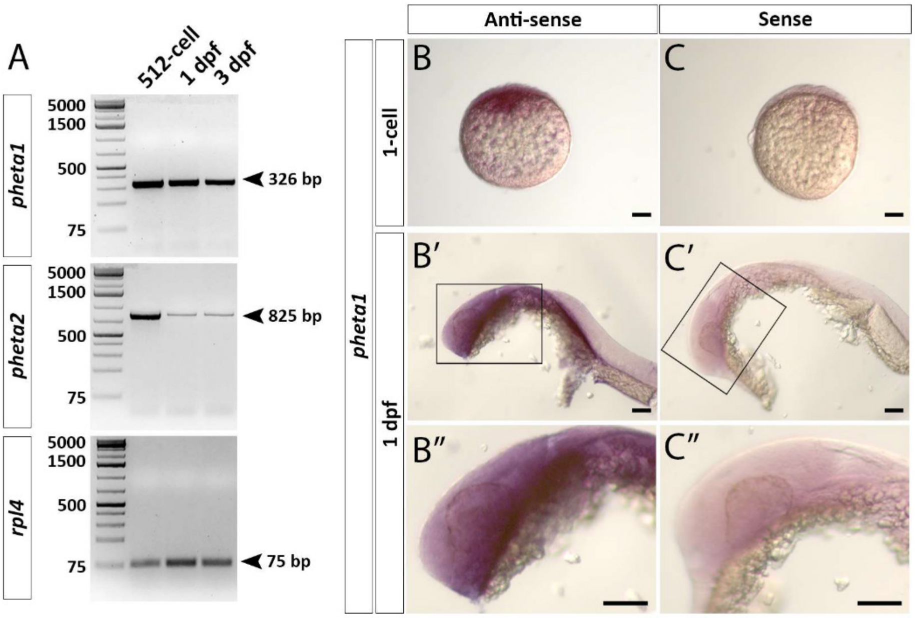

Figure S1. *pheta1* and *pheta2* transcripts are maternally inherited and expressed during development. (A) RT-PCR for *pheta1, pheta2,* and *rpl4* (a housekeeping reference gene) was performed from total RNA extracted from wild-type 512-cell, 1 dpf, and 3 dpf zebrafish. *pheta1/2* expression was detected in all stages, with the amount of transcript being the highest at the 512-cell stage, indicating high levels of maternal expression. DNA ladders are shown in the left-most lanes. DNA band sizes are as indicated by arrowheads. (B-C'') Whole-mount *in situ* hybridization for *pheta1* at the 1-cell and 1 dpf stage. Hybridization with the anti-sense probe (B-B'') showed that *pheta1* is expressed maternally (1-cell) and during development (1 dpf). The sense probe serves as negative control (C-C''). Boxed regions in B' and C' are shown in higher magnification in B'' and C'', respectively. Scale=100 μm.

Disease Models & Mechanisms • Supplementary information

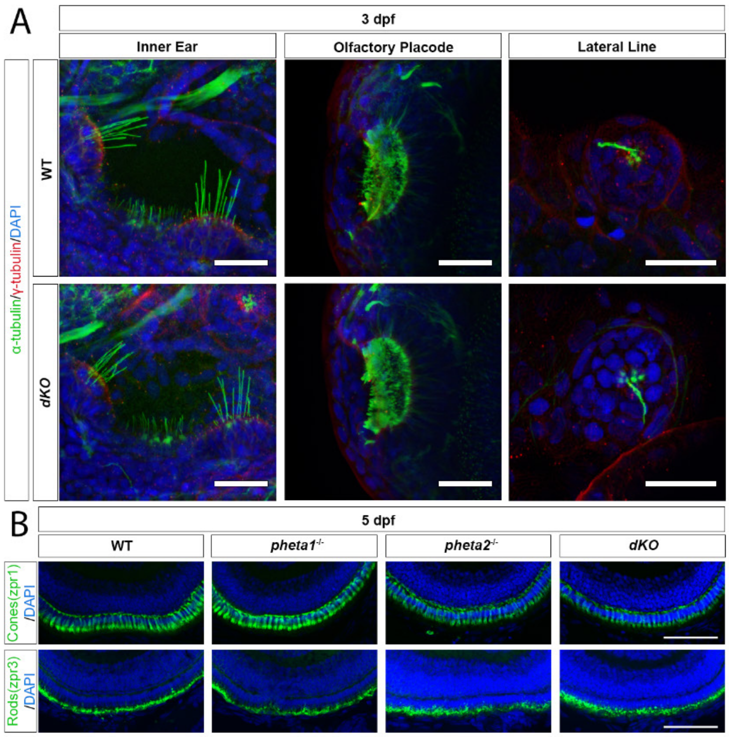

Figure S2. Loss of *pheta1/2* has no effect on ciliogenesis in the inner ear, olfactory placode, lateral line, and photoreceptors. (A) Representative confocal images of cilia in WT and *dKO* in the inner ear, the olfactory placode, and the lateral line. Cilia are labeled with anti-acetylated α-tubulin (green), basal bodies labeled with anti-γ tubulin (red), and nuclei labeled with DAPI (blue). Scale bar=25 μm.(B) Zpr1 (green) and zpr3 (green) antibodies were used to label cones and rods, respectively. Scale bar=50 μm.

Disease Models & Mechanisms • Supplementary information

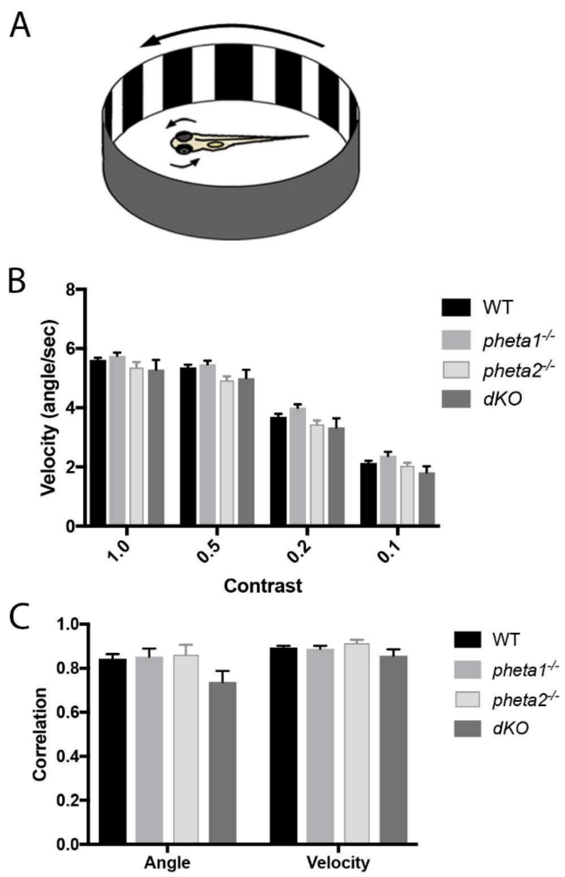

Figure S3. Loss of *pheta1/2* has no effect on OKR. (A) A zebrafish larva is placed in an arena with moving black and white gratings. An infrared camera records the position and speed of each eye during the visual stimulation. (B) Velocity of tracking movements in response to moving gradients at various contrasts at 5-6 dpf. (C) Correlation in angle and velocity between left and right eye. Error=SEM.

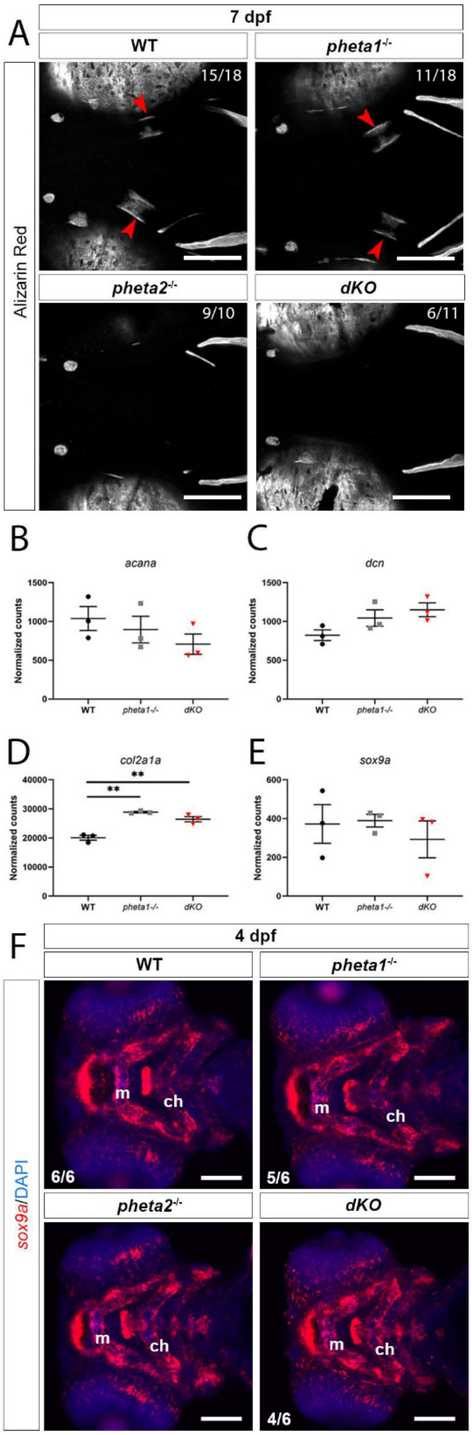

Figure S4. Ceratohyal ossification and chondrogenesis marker analysis. (A) Alizarin Red stained larvae, viewed from the ventral side. Bilateral bone collars in WT and *pheta1-/-* animals are indicated by arrowheads. The majority of *pheta2-/-* and *dKO* animals lack bilateral bone collars at this stage. The number of animals imaged with the displayed phenotype is shown in the upper right corner of each image. (B-E) RNA-seq normalized transcript counts for *acana* (B), *dcn* (C), *col2a1a* (D), and *sox9a* (E) at 5 dpf. **:$p<0.01$. (F) Fluorescent *in situ* hybridization for *sox9a*. Images show confocal maximum intensity projections, viewed from the ventral side. The number of animals imaged with the displayed phenotype is shown in the lower-left corner of each image. Error bar=100 μm. Abbreviations: ch, ceratohyal cartilage; m, Meckel's cartilage.

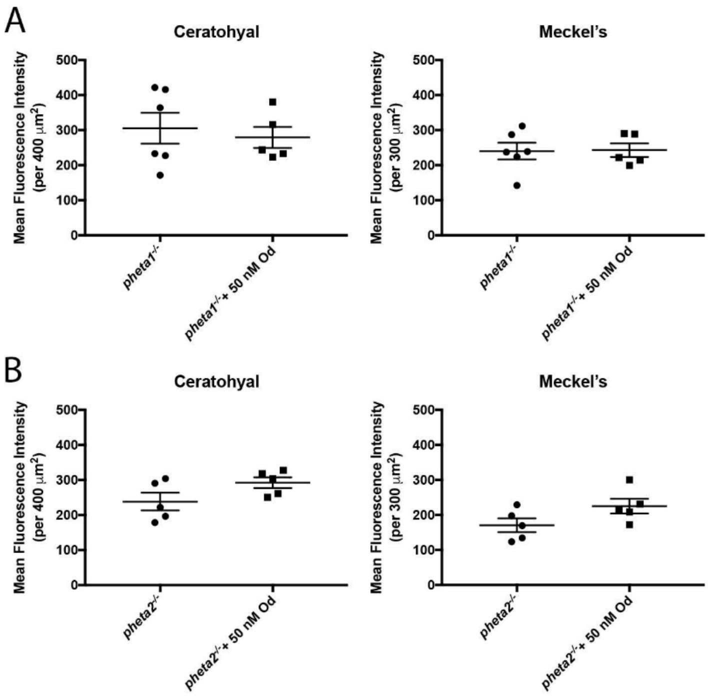

Figure S5. Col2 levels after administration of Od.

Quantification of mean immunostaining fluorescence intensity in the ceratohyal and Meckel's cartilage for *pheta1*$^{-/-}$ animals (A) and *pheta2*$^{-/-}$animals (B) at 4 dpf.

Disease Models & Mechanisms • Supplementary information

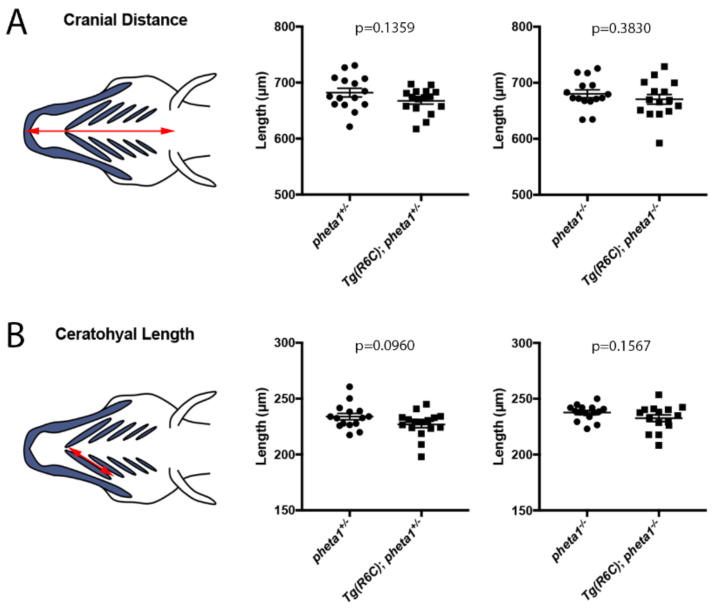

Figure S6. The effects of *Tg(R6C)* on cranial distance and ceratohyal length. Cranial distance (A) and ceratohyal length (B) in *pheta1*$^{+/-}$ and *pheta1*$^{-/-}$ backgrounds at 6 dpf, with and without the *Tg(R6C)* transgene. Measured structures are shown in the schematics on the left. *t*-test p values are as shown above graphs.

Disease Models & Mechanisms • Supplementary information

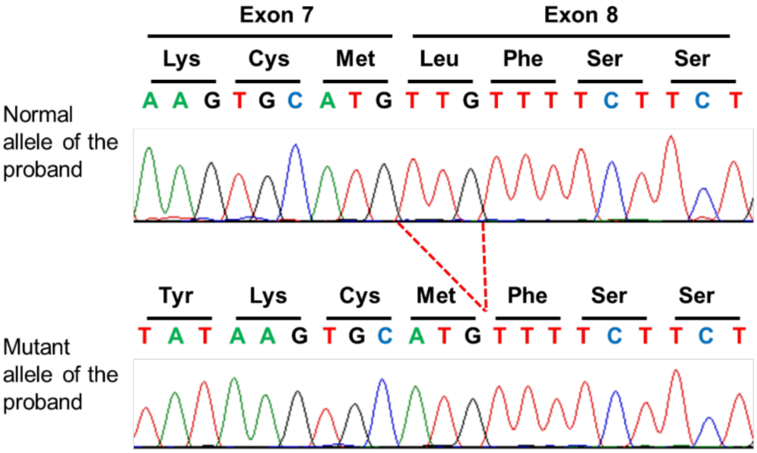

Figure S7. Splice site analysis of the *PHF6* variant in the UDP patient. Sequence chromatograms showing the normal allele (upper panel) and mutant allele (lower panel) of the UDP patient (i.e., proband). There was no splice defect except the in frame deletion of Leu244 at the exon 7 and 8 boundary (marked in red).

Disease Models & Mechanisms • Supplementary information
